# An alternative developmental table to describe non-model fish species embryogenesis: application to the description of the Eurasian perch (*Perca fluviatilisL.* 1758) development

**DOI:** 10.1186/s13227-015-0033-3

**Published:** 2015-12-18

**Authors:** Maud Alix, Dominique Chardard, Yannick Ledoré, Pascal Fontaine, Berenice Schaerlinger

**Affiliations:** UR AFPA, Université de Lorraine-INRA, 2 Avenue de la Forêt de Haye, BP 172, 54505 Vandœuvre-lès-Nancy, France

**Keywords:** *Perca fluviatilis*, Developmental table, Histology, Embryogenesis description, Digestive system ontogeny, Visual system ontogeny

## Abstract

**Background:**

Fish correspond to the most diversified phylum among vertebrates with a large variety of species. Even if general features are distinguishable during the embryogenesis, several differences in term of timing, organ implementation or step progression always occur between species. Moreover, the developmental timing of wild non-model fish often presents variability within a species. In that context, it is necessary to define a model of developmental table flexible enough to describe fish development by integrating this variability and allow intra- and inter-specific comparisons. The elaboration of a model passes by the definition of new stages that could be easily observable on individuals. The present study aims at proposing such a model and describing accurately the Eurasian perch (*Perca fluviatilis*) embryogenesis using microscopic techniques among which time lapse video and histological studies. The Eurasian perch belongs to the *Percidae* family that includes 235 species classified in 11 genera. It is a member of the *Perca* gender and inhabits the Northern part of Europe and Asia.

**Results:**

At 13 °C, *P. fluviatilis* development elapses for 15 days from the fertilization to the first oral feeding. The staging division first took into account the cellular status to define periods, then the acquisition of new abilities by the embryo to further define stages. It allowed distinguishing two main stages during the cell cleavage period depending on the synchronization of the cell divisions, two stages during the gastrulation period depending on the cell speed migration and five stages during the organogenesis according to the acquisition of key abilities as proposed in the saltatory theory. During each stage, organs implementation was carefully followed with a particular attention for the visual and digestive systems. In addition, our study shows that embryos hatch at various developmental stages while they all begin to feed at a fixed date, 15 days after the fertilization whatever the spawn and the hatching date. These data give arguments to propose the first oral feeding as the best definition of the embryonic-to-larval transition.

**Conclusions:**

The present model of developmental table combines flexibility and accuracy allowing detailed description of non-model fish species and intra- and inter-specific comparisons.

**Electronic supplementary material:**

The online version of this article (doi:10.1186/s13227-015-0033-3) contains supplementary material, which is available to authorized users.

## Background

Fish species have colonized numerous ecological niches allowing them to evolve and be adapted to various environments. As a consequence, fish represent the most diversified vertebrates regarding their lifestyle, shape, physiology, reproduction or development to quote only them. In that context, numerous developmental tables of various fish species have been published (e.g., the following recent studies 1–5). Besides common features, numerous differences emerge between fish species making difficult the standardization of embryogenesis staging. Two main description ways of the fish embryonic development co-exist. The first way mainly follows continuous morphological modifications of the embryos as for zebrafish, medaka or goldfish, for example [1, 5, 6]. Each step is accurately identified and some precise morphological criteria as the somites differentiation are used as the time scale of the organogenesis period [e.g., 6]. These fish are model species and represent teleosts fish in the developmental biology field. The description of their development needs to follow some accurate standards for comparative biology. However, these fish were mainly chosen as model for practical reasons as their ease of rearing in laboratory conditions, their short developmental period, their size or access to genetic tools compared to other fish species. Furthermore, they are well adapted to their rearing conditions and their development is very regular, allowing an acute repeatability in term of timing in controlled conditions. Nevertheless, the embryogenesis progress of non-model species is often less repeatable at a specific temperature [7]. For example, during the cell cleavage of the walleye, authors mentioned from the beginning that they observed individual variations leading to different number of blastomere whatever the time [8]. In addition, the mechanisms regulating somitogenesis and other organs ontogeny may not be synchronous in all individuals, rendering such model of developmental table quite difficult to apply.

Another way to describe fish development takes into account these variations allowing more flexibility. Balon [9] observed that fish embryogenesis progress by fits and starts. He thus proposed that ontogeny can be divided into two kinds of stages named thresholds and steps [9, 10]. Thresholds allow describing the sudden acquisition of a new ability. Their sequence highlights key stages allowing individuals to reach a new developmental level. During steps, slow and continuous development of several organs is observed before that sudden acquisition of a new ability and thus a new threshold. This approach permits a better description of the common features observed in various fish species while taking into account potential intra and inter-specific variations. However, in the studies performed with this model, fish development is often less accurately described.

Instead of opposing these two schools, it would thus be more profitable for scientists to take into account advantages of each model and improve accuracy while facilitating intra and inter-specific comparisons of fish development. To do so, it would be interesting to trace the accurate description of organs ontology performed in the first model on a time scale different from somitogenesis. In this condition, the definition of steps and thresholds as a new time scale would be more flexible. The present work aims at describing organs ontogeny in order to define precisely steps and thresholds and make an accurate timing of perch early development.

Another definition opposing scientists is the question of the embryonic-to-larval transition [11]. Indeed, while it is commonly admitted that the starting point of the embryogenesis corresponds to the egg activation occurring at the ova releasing in the water [9, 12], the end of the embryogenesis is defined either by the hatching or the first oral feeding depending on the authors [for review 9, 12, 13]. From a general point of view, the first definition of an embryo corresponds to a specific period of the development during which individuals are protected by an envelope [12, 13] or a gelatinous layer surrounding the embryo that is called gangue [14]. In the meantime, larva mainly defines the developmental period elapsing from the hatching to the juvenile stage often characterized by the metamorphosis. These definitions are used for several animal taxa (e.g., insects, molluscs, amphibians, etc.). In addition, several authors argue that hatching corresponds to an important stage because individuals are suddenly subjected to a new environment defined by physico-chemical parameters (e.g., pH, oxygen, etc.) different from those they got used. These sudden modifications force embryos to change their behavior and physiology [12, 13]. On the other hand, other scientists argue that embryos are only dependent upon reserves accumulated in the egg during oogenesis while the larval period corresponds to a step during which animal development needs external supply of energy, nutrients and material to continue. The embryonic period, then, allows the animals to acquire full abilities to chase their preys or protect themselves from predators at larval stages. In that context, the embryonic-to-larval transition rather takes place during the first oral feeding [9]. In comparison, hatching mostly corresponds to a long period within a spawn, while the first food intake occurs during a narrower timing for a numerous number of fish species although this statement is not common for every fish species because some of them display synchronous hatching [13, 15]. However, defining the first oral feeding as the embryonic-to-larval transition implies to make a difference between the development before and after hatching. In these conditions, individuals developing outside the protective chorion are defined as “free embryos” [9].

The present study aims at describing Eurasian perch (*Perca fluviatilis*) embryogenesis. It is a temperate freshwater fish species that inhabits from Northern countries of Europe to the North-East Asia, with some introduced populations in Southern countries of Europe and some countries of the South hemisphere [16, 17]. Its flesh is appreciated and the Eurasian perch belongs among the most promising fish species to promote freshwater fish diversification [18–20]. Perch domestication is still under process and its reproduction has already been investigated allowing a good knowledge of its reproduction cycle [18, 20–27], egg quality [28, 29] and larval development [30]. However, only few studies investigated perch embryonic development [31–33]. In 1854, Lereboullet performed the first description that was quite precise from the fertilization to the disappearance of the yolk. The author divided perch embryogenesis into four phases: (1) cell cleavage and gastrulation, (2) from the beginning of the organogenesis to the first heart beatings, (3) from heart beatings to hatching and (4) from hatching to the yolk disappearance [31]. He focused his description on the circulatory and intestinal systems. Later, in 1925, Chevey [32] mainly focused his attention on the cell cleavage and the gastrulation but poorly described organs differentiations until hatching. In the lab, a preliminary study allowed us to determine large periods of perch development [33]. Eurasian perch belongs to the Percidae family that is divided into 11 genders: *Ammocrypta*, *Crystallaria*, *Etheostoma*, *Gymnocephalus*, *Perca*, *Percarina*, *Percina*, *Nothonotus*, *Romanichthys*, *Sander* and *Zingel*. With the American yellow perch (*Perca flavescens*) and the Balkhash perch (*Perca schrenkii*), they compose the *Perca* gender. Its reproduction cycle lasts for about 9–10 months and its natural spawning season begins when the water temperature reaches around 12 °C [21] and elapses from mid-March to June depending on the geographical localization. Perches are group-synchronous fish meaning that their gonadogenesis is synchronous and that one female spawns at one time all its eggs [25]. Its gonadogenesis is under the control of the water temperature and the photoperiod. Recent studies aimed at developing a photothermal program allowing obtaining spawn in artificial controlled conditions (indoor) and even out-of-season reproduction [34]. However, it appears that the reproduction success is highly variable with important developmental impairments during early embryogenesis (personal observations). To better understand the reasons and the consequences of these developmental defects, it is essential to better characterize *Perca fluviatilis* early development and define a developmental table as a reference.

In order to describe accurately the Eurasian perch embryogenesis, time lapse video and histological techniques were used. Data show that while hatching can elapse for 5 days and that individuals hatch at different developmental stages, all embryos first feed 15 days after the fertilization whatever their hatching dates. These data suggest that the first oral feeding rather represents a threshold as defined by Balon [9] and thus is more accurate to define the embryonic-to-larval transition. In these conditions, the total duration of *P. fluviatilis* embryonic development elapses for 15 days at 13 °C, from the fertilization to the first oral feeding. The first main division of the development was performed according to the cellular state (cell division, migration or differentiation) to separate zygote, cell cleavage, gastrulation and organogenesis periods. Those periods were then further cut into steps separated by well-defined thresholds. In addition, we focused a particular attention to the digestive and visual systems ontogeny throughout embryogenesis because they play an important role, later, during larval stages.

## Methods

### Origin of fish, broodstock

Fishes were handled in accordance with national and international guidelines for protection of animal welfare (Directive 2010/63/EU). The natural spawning season of *P. fluviatilis* elapses from mid-March to the end of May in the East part of France (Lorraine). In order to have an accurate timing of the Eurasian perch development’s progress, three independent experiments have been conducted on fish with diverse origins in 2013, 2014 and 2015. During the first season (April–May 2013), the experiment was performed on two groups of breeders: (1) fish (Geneva Lake origin) provided by fish-farm “Lucas Perches” (Hampont, France) that were induced in tanks using the photo-thermal program [34] (Population 1) and (2) perch caught in ponds (provided by the GAEC Piscicole du Saulnois, France) (Population 2). During the second season (March–April 2014), three groups of breeders were used. Two populations were caught in ponds and provided by the GAEC Piscicole du Saulnois and the GFA du Kuhweg (Lorraine, France) (Populations 3 and 4). The third one (Geneva Lake origin) was provided by the intensive fish-farm “Lucas Perches” (Hampont, France) (Population 5). Their reproduction cycle was induced in our indoor facilities in 500 L according to Fontaine et al. [34]. During the third season (October 2015), one group of breeders originating from the Geneva Lake and provided by the fish-farm “ASIALOR” (Dieuze, France) was used (Population 6). They were induced in tanks using the photo-thermal program [34]. Breeders from the first population were kept in indoor tanks and submitted to artificial conditions all along the reproduction cycle and the spawning season. Breeders caught in ponds (Populations 2, 3 and 4) were transferred 1 month before the natural spawning season, in our outdoor facilities consisting of a recirculating system (tanks of 3000 L, Laneuvelotte, Lorraine, France). Breeders from the fifth population were also transferred from indoor tanks into our outdoor facilities before the spawning season. Breeders from the sixth population staid in the fishfarm and eggs ribbons from four females were collected and transferred in our indoor facilities 6 days after the fertilization.

### Hatcheries management and water quality

Each incubator consists of a water recirculating system of 1400 L with a flow rate of 18 m^3^/h. They were under municipal water system and air and water’s thermoregulation was maintained with air-conditioning and refrigerating unit (2200 W). In the first incubator, used for the daily following of the embryonic development, egg ribbons (17,000–84,000 eggs) were transferred in large crates allowing aeration thanks to a continuous horizontal water current in each crate. For large spawn several crates were used per spawn. In the second incubator, three samples of ribbons (around 100 eggs/sample) for each spawn were kept in baskets until hatching to determine hatching time and duration. Baskets were aerated thanks to the water flow. So, for every spawn, some embryos were incubated in each of the incubators. This division was done to optimize the space in hatcheries and facilitate our organization. No difference of hatching timing and duration was observed between samples of one spawn in the different incubators. The optimal water temperature for perch development has been defined as 13 °C [35]. In the present work, the mean temperature was kept at 13.0 ± 0.5 °C in all hatcheries during each season. A constant dark/light photoperiod (8:16) was applied with two neon lamps at a light intensity of 200 lx at the water surface. Water levels of pH (8.19 ± 0.09), dissolved oxygen (9.84 ± 0.16 mg/L), nitrite (0.16 ± 0.16 mg/L) and ammonium (0.17 ± 0.17 and 1.08 ± 0.95 mg/L, in 2013 and 2014, respectively), were monitored twice a week in breeders’ tanks (indoor and outdoor) and in the hatcheries. In order to prevent bacterial or fungal infections, experimenter used footbath, antibacterial solution for hand and the water was sterilized with UV.

### Gametes collection and fertilization

*Perca fluviatilis* has a group-synchronous oogenesis but within a population all females don’t spawn exactly at the same time and thus the spawning period can elapse from 3 to 5 weeks within a broodstock. The beginning of the spawning season was considered as triggered once the first ribbon was observed in a tank. From that time, each perch was caught daily to check specifically its reproductive status. Once a female was mature (apparition of a blister at the genital orifice), it was stripped to collect ova in dry glass beakers. Their activation was done for 15 s by the addition of water from breeders tanks [20]. Subsequently, in vitro fertilizations were performed by adding to the ova a mixture of sperm of three different males in order to optimize the fertilization success.

### Study of the developmental progress by microscopy and time lapse microscopy

Fish early embryogenesis was followed by time lapse microscopy on embryos originating from 13 spawn (total number of records = 54) from the first cell cleavage (2.5 hpf) to the beginning of the organogenesis (99.5 hpf) when the muscle contractions prevented to perform properly the time lapse experiment. The increase of water temperature under the microscope can modify the developmental speed of the embryos observed in comparison to those from the same spawn staying in incubators. In order to avoid bad recording of the developmental timing, embryos were removed after 1 h of development. Then, new embryos from the hatchery were transferred into a Petri dish filled with fresh water and placed under the microscope. In this condition, the developmental timing recorded, was respecting the one observed in the incubator. The time lapse microscopy was performed using a light upright optical microscope (Nikon Eclipse Ni-U) associated with a DS-Fi2 digital camera and the software NIS BR (Nikon France, Champigny-sur-Marne, France). A picture was taken every 40 s or every minute depending on the process velocity. Pictures were taken at low magnification (2×).

From day three post-fertilization, embryos from 36 spawn have been observed to describe organogenesis. Among these spawn, 13 were submitted to a careful daily following (*n* > 3 embryos/spawn/day). Embryos were sampled once or twice a day for microscopic observations. The jelly coat was removed using micro-dissecting forceps under a binocular microscope. In order to create all-in-focus pictures for some samples, we used the “extended depth of focus” (EDF) option of the software. Moreover, large images were taken thanks to “grab a large image” option of the software. Pictures were taken at low magnification (2×). The embryonic total length was measured daily on fixed embryos (3 spawn, 15 embryos/spawn/day) using the Nikon BR software. Data are presented as a mean value ± standard deviation.

### Fixation and histology

Embryos were sampled once a day from 1 to 15 dpf and fixed in Bouin’s solution for 48 h before being washed and conserved in 70 % alcohol. In order to facilitate their cutting orientation, embryos were first placed in 1.5 % agarose blocks. These blocks were then dehydrated in ascending series of ethanol (70–100 %) before being embedded in paraffin. Four micrometer sections were performed with a Leitz Wetzlar microtome and collected on glass slides. Hematoxylin and Eosin staining were performed according to a protocol adapted from Gabe [36] as follows: Eosin Y (Sigma-Aldrich, Saint Quentin Fallavier, France) was diluted in water at 1 % and used from 30 s to 1 min; Hematoxylin solution modified according to Gill III (Merck, Darmstadt, Germany) was used from 5 to 10 min. Stained sections were examined using a light upright optical microscope (Nikon Eclipse Ni-U) at 10× and 40× magnifications (Nikon France, Champigny-sur-Marne, France). In total, 45 embryos from 11 spawn have been used for the histological study. Three embryos were transversally cut per day from 4 to 15 dpf. In addition, three embryos were longitudinally cut at 5, 10 and 15 dpf.

### Determination of the first oral feeding period

Two independent experiments were used to determine the first oral feeding (one in 2014 and the other one in 2015). In the first one, fifty embryos randomly chosen from six spawn were used and in the second one four spawn (*n* = 4–10 for each condition). The hatching period was checked daily until the first hatching. Each day of hatching all embryos were taken and put in a new crate. Hatching dates elapsed from 8 to 14 dpf depending on the spawn for the first experiment and from 9 to 14 dpf for the second one (e.g., for the second experiment, spawn 1 hatched between 11 and 13 dpf; spawn 2 between 9 and 12 dpf; spawn 3 and 4 between 9 and 14 dpf). All spawn were kept and studied in the same conditions within one experiment. Embryos were taken at their date of hatching and kept in 1–2 L beakers in the first experiment or in 50 mL tubes in the second experiment. This difference of methodology is due to technical reasons in our hatcheries. In every case, containers were filled with the water of the hatchery and plunged in the hatchery to maintain the water temperature around 13 °C. Each container corresponds to one group of embryos coming from the same spawn and that hatched at the same dates. So for one spawn, there was as much container as hatching dates and each spawn was independently studied in both experiments. In the beakers, the volume of water was large enough to allow oxygenation for several hours. The 50 mL tubes contained 40 mL of water. No lethality was observed while embryos were in the tubes suggesting that there was no problem of oxygenation. Rinsed alive *Artemia* nauplii (Catvis, size: EG) were supplied as food to embryos daily from hatching (20 mL; density = 10–14/mL). After 2 h with *Artemia*, the digestive system of alive free embryos was observed using binocular Olympus (SZX7) at low magnification (2×). As embryos are transparent, the presence of *Artemia* was easily detectable in the digestive tract. If the embryos did not eat, the water was changed in the beakers to perform another time the experiment in the experiment one. In the experiment two, new embryos taken from the same crate were used to continue the experiment. Once embryos ate, they were submitted to an overdose of Tricaine methanesulfonate (200–300 mg/L; Sigma-Aldrich) until they die according to the European Ethical guidelines (Directive 2010/63/UE).

### Comparison of the developmental process between hatched and unhatched embryos

The goal of this experiment was to clarify whether embryos that hatch early developed with the same speed than those of the same spawn that still remained in the envelope. The experiment was performed on four spawn from the population 6. The developmental process was assessed thanks to the apparition of 12 easily observable criteria as followed from the earliest to the latest: the presence of melanophores on the body, the eyes pigmentation, the circulation in the yolk and later on the ventral part of the embryos, their straightening, the apparition of the urinary bladder, the bile, the beginning of the peristaltic undulations, the observation of coordinated fins movements and later the jaw quivering, the mouth opening and finally the first oral feeding. In addition the size of naturally hatched embryos was measured at low magnification (2×) from their hatching to the first oral feeding (*n* = 3 spawn). Data were treated according to [37] using the Nikon BR software and are presented as a mean value ± standard deviation. During the hatching period, embryos were monitored daily to determine their hatching dates. Three newly hatched embryos per spawn were sampled everyday and isolated in small cages kept in the hatchery until their first oral feeding. In this manner, it was possible to follow the progression of their development of every individual from their hatching date. Everyday, from the first hatching, a sample of three embryos per spawn that were still embedded were removed from the gangue with forceps. Once they were observed, these embryos were removed from the experiment and a new batch of not yet hatch embryo was freed to be used the next day. Three categories of embryos were distinguished: NHE for newly hatched embryos corresponding to their state when the naturally hatched. Later, they became FE (free embryos) that corresponds to the same embryos but from the day after their hatching. Finally, UE correspond to unhatched embryos that were removed from the envelope with forceps. All embryos were observed under a light upright optical microscope (Nikon Eclipse Ni-U) at 2×, 4×, 10× and 40× magnifications (Nikon France, Champigny-sur-Marne, France). The apparition of each criterion was recorded daily for each embryo until they first feed. The comparison between the newly hatched embryos and their not yet hatched counterparts was done to determine whether there were differences of developmental rate once the embryo leave its envelope.

### Statistical analyses

The water quality parameter data are presented as mean ± SD. The normality and homogeneity of variances were tested using a Levene test. A one-way analysis of variances (ANOVA) was performed with one factor (year of experiment). The minimum level of significance was set at *p* < 0.05. When data did not respect the assumptions of normality, a Kruskal–Wallis test followed by a non-parametric Mann–Whitney test were performed.

For the morphometric measurement performed to determine the size difference between free and embedded embryos, data are presented as mean ± SD. The normality and homogeneity of variances were tested using a Levene test. A two-way analysis of variances (ANOVA) was performed with two independent factors (factor 1 = groups NHE, UE, FE and factor 2 = time) followed by Bonferroni’s post hoc tests to determine significant differences. The minimum level of significance was set at *p* < 0.05. When data did not respect the assumptions of normality, a Kruskal–Wallis test followed by a non-parametric Mann–Whitney test were performed. All the statistical analyses were performed using STATISTICA 12.0 program (Statsoft Inc., Tulsa, OK, US).

## Results

**Fig. 1:**
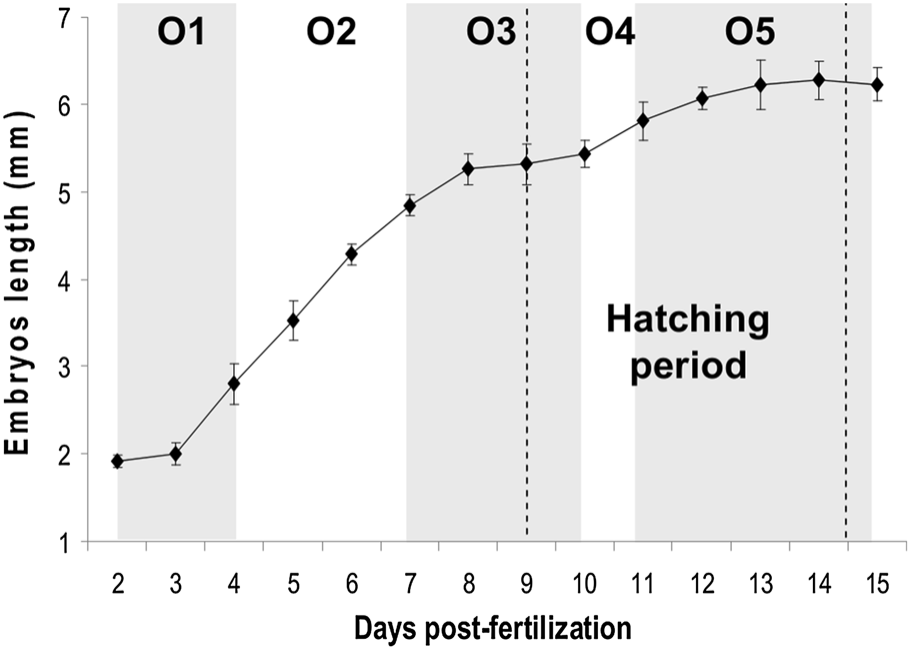
Embryonic total length during the organogenesis period. Every day 15 embryos/spawn were measured (three spawns). Data correspond to the mean value of the three different spawns ± SD. The five organogenesis periods are given for information (O1 to O5) and the peak of the hatching period is presented between the *dotted lines*

Fish embryogenesis begins when the ova is activated, allowing the fertilization and ends at the first oral feeding. It lasts for 15 days at a constant temperature of 13. ± 0.5 °C (195°d). The mean embryonic total length increases from 0.98 (zygote diameter) to 6.23 mm at the time of the first exogenous feeding (Fig. 1). The embryonic growth is fast during the first steps of the organogenesis period and slows down before the hatching period until the end of the study. In the present work, the starting time corresponds to the fertilization.

### The zygote period (0–2.5 hpf)

**Fig. 2:**
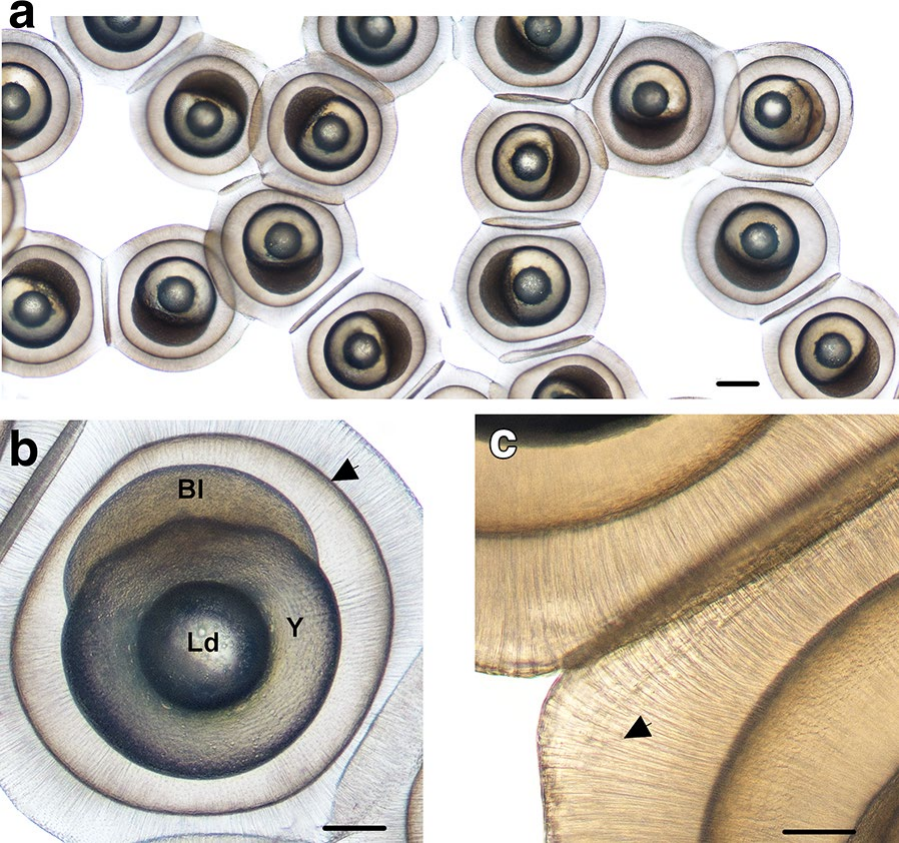
Morphological characterization of *P. fluviatilis* spawn and zygote. **a** Perch embryos embedded in the jelly coat allowing forming a ribbon of eggs. The *scale bar* represents 500 µm. **b** Face view of zygote stage. *Bl* blastoderm, *Y* yolk, *Ld* lipid droplet. The chorion (*arrowhead*) surrounds the zygote. The *scale bar* represents 250 µm. **c** Detail of jelly coat presenting tubular structures (*arrowhead*). The *scale bar* represents 100 µm

The zygote is surrounded by a thin chorion (Fig. 2b, arrowhead) and a large gelatinous envelope (or jelly coat) organized with tubular structures (Fig. 2c) called *zona radiata externa* as previously described by Formicki et al. [14]. It allows maintaining the eggs together and forming a ribbon (Fig. 2a). After the fertilization, the mean zygote diameter is 0.98 ± 0.065 mm, without the jelly coat. A lipid droplet is present on the vitelline reserve (Fig. 2b) that is most of the time unique but can sometimes be divided into several smaller droplets. At the animal pole, the cytoplasm separates from the yolk to form the blastodisc.

### The cell cleavage period (2.5–24 hpf)

**Fig. 3:**
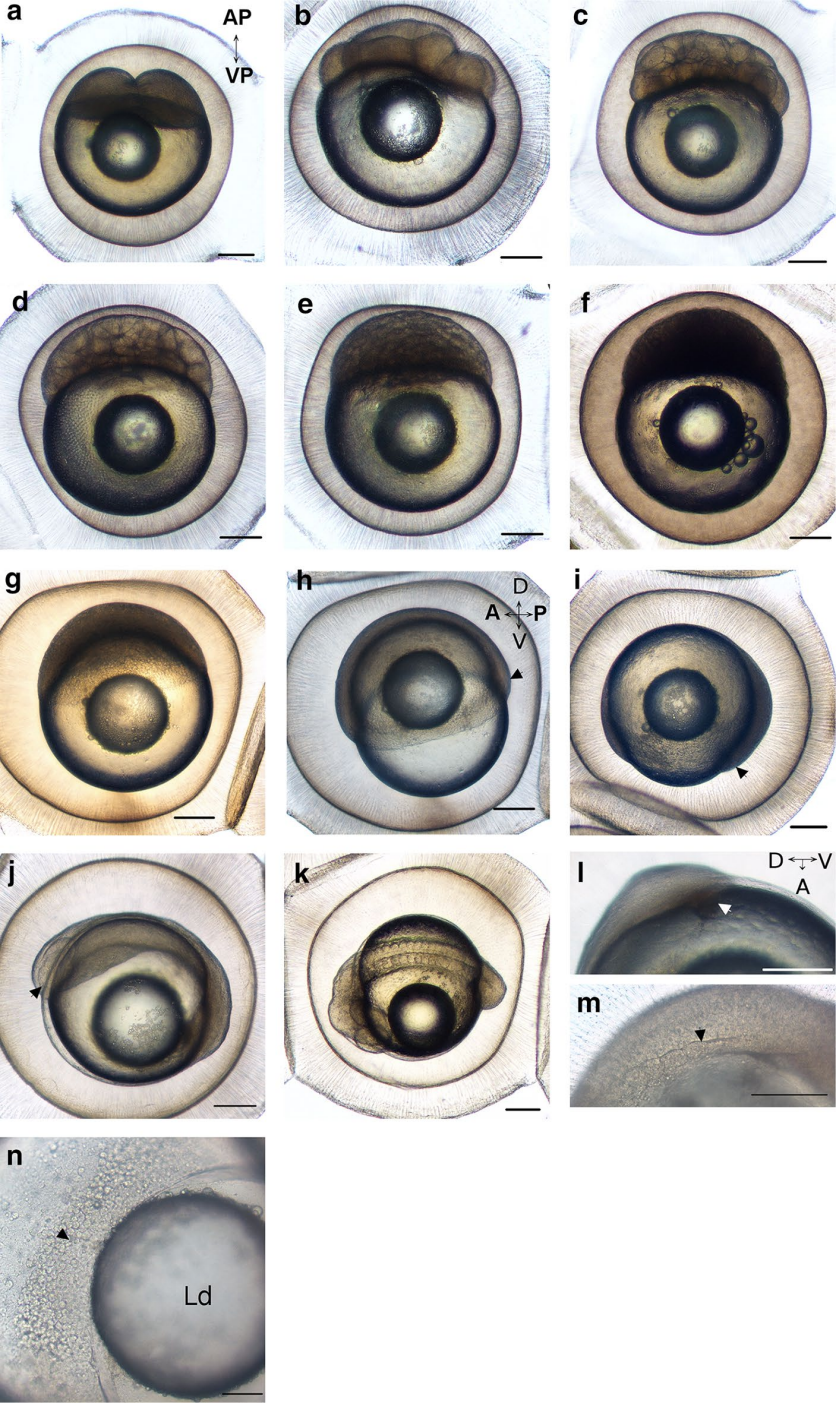
Cell cleavage and gastrulation periods. **a**–**j** Face view of **a** two-cell stage (3.25 hpf) at the animal pole of the embryo (*AP* animal pole, *VP* vegetative pole). **b** Height-cell stage (5.5 hpf), **c** 32 cells stage (8 hpf), **d** 128 cells stage (9.5 hpf), **e** 512 cells stage (11.5 hpf), **f** high blastula (14.25 hpf), **g** 30 %-epiboly stage (25.75 hpf), **h** 50 %-epiboly stage (31.25 hpf). The antero-posterior AP and dorso-ventral DV axes can be determined for the first time. The *arrowhead* highlights the posterior part of the germring. **i** 90 %-epiboly stage (40.25 hpf). The *arrowhead* highlights the posterior part of the germring. **j** Embryo at the beginning of the somitogenesis (4–6 somites, 64 hpf), Embryonic axes are well defined, the *arrowhead* shows optic capsules. **k** Embryo during the O1 period. (81 hpf). **l** Detail of Kupffer’s vesicle (*arrowhead*), (52.75 hpf). **m** Detail of the somites (*stars*) (64 hpf). **n** The yolk syncytial layer with numerous nuclei (*arrowhead*) (Ld) lipid droplet. The *scale bar* represents 250 µm (**a**–**m**) and 100 µm for **n**

The first cleavage (2.5–4 hpf) follows the animal to vegetative poles axis and results in two large cells of equivalent size (Fig. 3a). Between 4 and 5 hpf, the second cleavage occurs perpendicularly to the first division but still in the animal to vegetative poles axis generating four blastomeres. The eight-cell stage occurs between 5 and 6 hpf by cutting the blastoderm perpendicularly from the animal to the vegetative pole in order to obtain two rows of four cells (Fig. 3b). The next cleavages cut the embryo either along the animal to vegetative axis or perpendicular to it (Fig. 3d–f). Surprisingly, divisions are accompanied by waving movements coming from the yolk to the animal pole that is strong during the first cell divisions (Additional file 1) but become less important later in the process (Additional file 2). This loss of yolk contractions is accompanied by an asynchrony of cell divisions appearing between 64 and 128 cells stage and later a slowing down of the cell division from the stage 512 cells (Table 1). These differences allow us to separate the cell cleavage period into two steps “cell cleavage 1” (CC1) and “cell cleavage 2” (CC2) for the synchronous and asynchronous steps, respectively. However, the cell division does not have the same rate for every embryo and within a spawn this period is not synchronous between neighbor embryos or between spawn, rendering difficult an accurate timing of this period. In spite of this heterogeneity, the end of the cleavage period is always at 24 hpf.

**Table 1:** Durations of cell cleavages from two cells to the high blastula stage

Stage	Mean duration (h)
2 cells	1.5
4 cells	1
8 cells	1
16 cells	1.5
32 cells	1
64 cells	1.25
*128 cells*	*1*
*256 cells*	*1.5*
*512 cells*	*3*
*1024 cells*	*2*

From 128 cells stage, the cell cleavage becomes asynchronous (italic)

### The gastrulation period (24–50 hpf)

The gastrulation is defined as the period during which cellular movements (involution, convergence and extension) of the blastoderm tend to form the embryonic axis and the organization of the germ cell layers: the epiblast that will give rise to a part of the ectoderm, the hypoblast that corresponds to the future mesoderm and endoderm [6]. First, epiboly movements begin at 24 hpf and the cellular front reaches 30 % of epiboly at 25.75 hpf (Fig. 3g; Table 2) and 50 % of yolk surface at 29.50 hpf (Fig. 3h; Table 2). At that stage, asymmetric thickening of opposite extremities of the embryos corresponding to the embryonic shield stage is observed, allowing the first visualization of the antero-posterior axis (Additional file 3). Until 70 % of epiboly, the cell progression along the yolk is regular and fast. It corresponds to the step gastrulation 1 (G1). During the second step (G2), from 70 % epiboly stage, the cellular front progresses more slowly. The germ ring compresses the yolk (31.25 hpf) and the posterior end of the embryo can be distinguished (Additional file 3). Later both extremities of the embryo continue to thicken even if the posterior part always remains thicker until the 90 %-epiboly stage (Fig. 3i, 40.25 hpf). The yolk syncytial layer (YSL) corresponding to a multinucleated cell layer between the yolk and the blastomeres is visible (Fig. 3n). This structure is observed later during embryogenesis. In contrast to the walleye [8], except for some rare cases, no embryonic rotation has been observed during perch gastrulation.

**Table 2:** Timing of the gastrulation and the somitogenesis

Stage	Apparition
30 % epiboly	25.75 hpf
50 % epiboly	29.50 hpf
70 % epiboly	31.25 hpf
80 % epiboly	36.25 hpf
90 % epiboly	40.25 hpf
Tailbud	51.75 hpf
Three somites	58.25 hpf
4–5 somites	64 hpf
12 somites	76.75 hpf
15 somites	77.25–81 hpf
17 somites	83.25 hpf
35–40 somites	8–15 dpf

Numbers correspond to the time by which the developmental process occurs

### The organogenesis period (50 hpf–15 dpf)

During this period, cells begin to differentiate in order to achieve organs implementation. Only key steps of the development of several organs from 3 to 15 dpf are described: organogenesis has thus been divided into five steps separated by well-defined thresholds. Then, the description of the digestive and visual systems developments is presented.

### Organogenesis subdivision

#### Organogenesis 1 (O1) stage from the first cell differentiation to the first heart beatings (2–4 dpf)

One recurrent cell differentiation stage is the somitogenesis that occurs sequentially from the trunk to the most posterior side of the embryo. At the beginning of this stage, embryos measure 1.92 mm ± 0.06 (Fig. 1). The optic capsule (Fig. 3j, arrowhead) appears at 50 hpf, before the tail bud closure (51.75 hpf) suggesting that the cell differentiation begins before the end of the gastrulation movements. The three first somites can be observed at 58.25 hpf on living embryos. At 64 hpf, 4 or 5 pairs of somites are visible (Fig. 3m, arrowhead; Table 2). Twelve pairs of somites are developed at 76.75 hpf. Between 77.25 and 81 hpf, depending on the spawn, 15 pairs of somites can be seen (Fig. 3k). Other organs progressively appear as the Kupffer’s vesicle that is observable from 52.75 hpf (Fig. 3l, arrowhead) to 84 hpf. As for the walleye [8] a translocation movement allowing decreasing the distance between embryo’s head and the lipid droplet occurs the third day of development. The notochord is first visible at 55.75 hpf on living embryos and begins to be vacuolated from the 4th dpf. At this stage, embryo’s length attains 2.80 ± 0.20 mm (Fig. 1) and the auditory vesicles begin to develop. The epithelium of the embryo appears and is composed by various cell types such as the mucous and the club cells and later in the organogenesis, by the keratinocytes. During the same day, at 99.5 hpf, the first heart beating and spontaneous muscle contractions were recorded. The heart is already composed of two separate cavities corresponding to the future ventricle and atrium. The tail begins to separate from the yolk.

#### Organogenesis 2 (O2) stage from the first heart beatings to the differentiation of the organs associated to the digestive system (4–7 dpf)

**Fig. 4:**
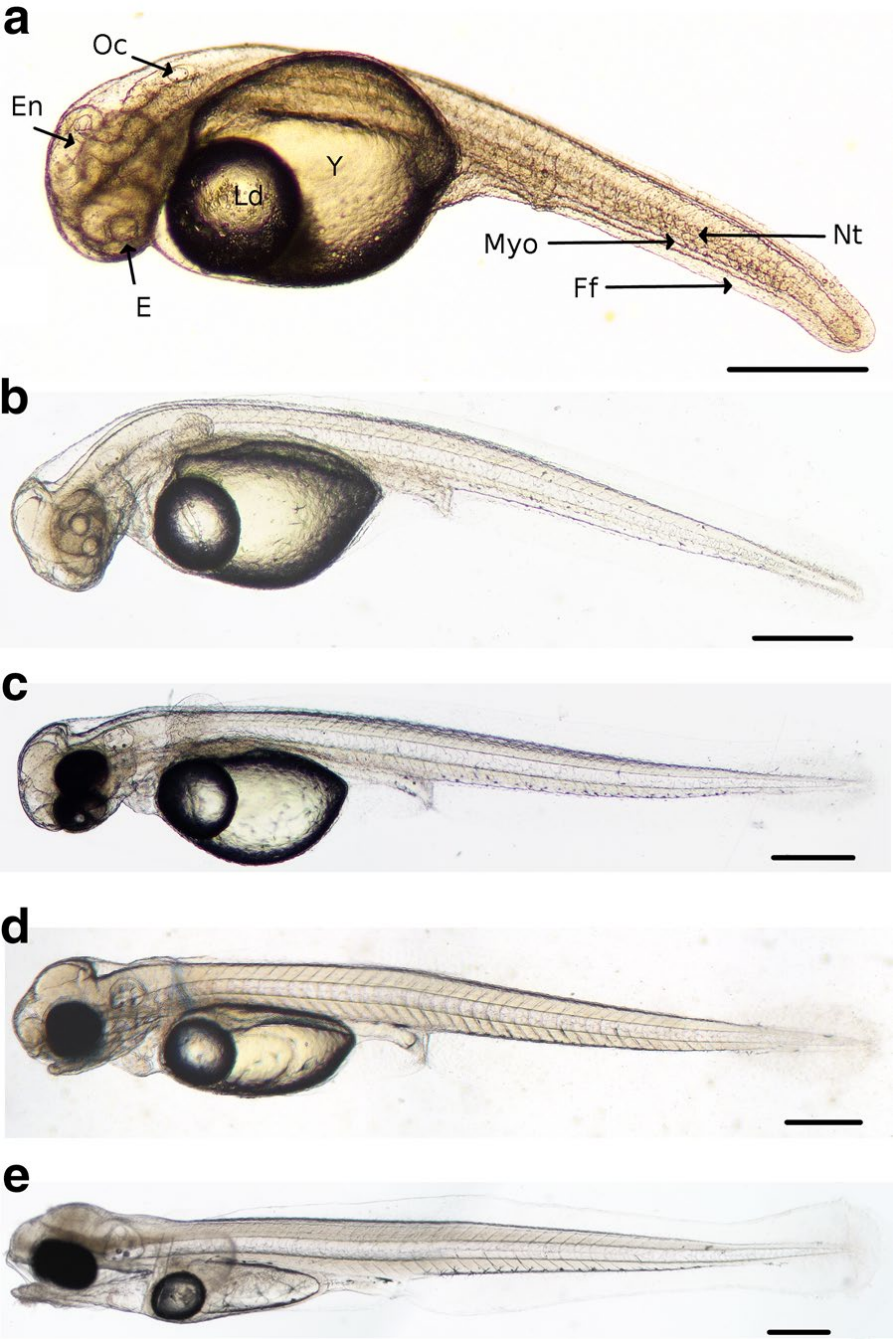
Comparison of the embryonic morphology during *P. fluviatilis* organogenesis. **a** 5 dpf living embryo representative of the O2 period. Several organs are already visible as the encephalon *En*, eyes *E*, lipid droplet *Ld*, yolk *Y*, myotome *Myo*, notochord *Nt* and the otic capsule *Oc*. **b** 7 dpf representative to the beginning of the O3 period. **c** 9 dpf representative to the end of O3 period. **d** 11 dpf, representative of the O4 period. **e** 15 dpf representative of the O5 period. *Scale bars* represent 500 µm

**Fig. 5:**
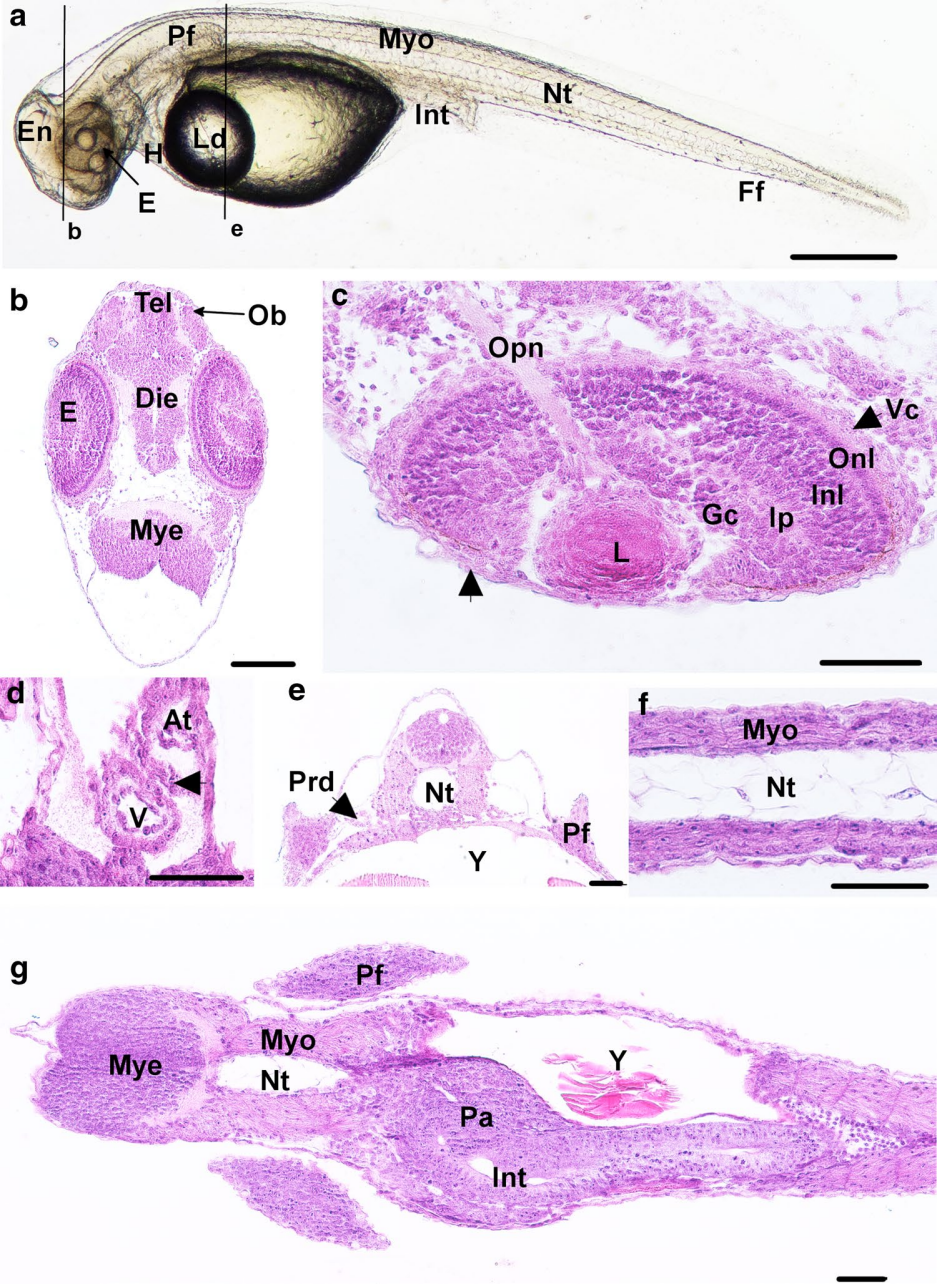
Detailed description of an embryo 7 dpf representative to the beginning of the O3 period. **a** Whole embryo. *Black lines* represent the location of transverse sections shown in **b**, **e**. **b**, **c**, **e** Transverse sections and **d, f**, **g** coronal sections. **b** Section of the head. **c** Detail of an eye. The *arrowhead* shows pigmented cells. **d** Detail of the heart. The *arrowhead* shows the atrioventricular valve. **e** Section of the trunk. **f** Vacuolated notochord surrounded by myotomes. **g** Coronal section of the head and the trunk. *At* atrium, *Dien* diencephalon, *E* eyes, *En* encephalon, *Ff* finfold, *Gc* ganglion cells, *H* heart, *Inl* inner nuclear layer, *Int* intestine, *Ip* inner plexiform, *L* lens, *Ld* lipid droplet, *Mye* myelencephalon, *Myo* myotome, *Nt* notochord, *Ob* olfactory bulb, *Onl* outer nuclear layer, *Opn* optic nerve, *Pa* pancreas, *Pf* pectoral fins, *Prd* pronephric ducts, *Tel* telencephalon, *V* ventricle, *Vc* visual cells, *Y* yolk. *Scale bars* represent 500 µm for **a** and 50 µm for **b**–**g**

At day 5 post-fertilization the brain is subdivided into three parts: (1) the forebrain (future telencephalon and diencephalon), (2) the midbrain (future mesencephalon and metencephalon) and (3) the hindbrain (future myelencephalon) (Figs. 4a, 5a). In addition, the olfactory bulb located in the most anterior part of brain (Fig. 5b) and the *medulla oblongata* in the myelencephalon are observed. The *medulla oblongata* forms a continuum with the spinal chord that follows dorsally the notochord all along the embryo. At 5 dpf, a pair of otoliths is clearly visible in the otic vesicles (Figs. 4a, 5a) and the first small patches of melanophores appear on the yolk. Pectoral fin buds are present behind the heart cavity, just above the vitelline mass. The cardiac cavity’s enlarging is observed at 6 dpf, in the same time than the onset of circulation on the ventral-posterior side of the yolk, behind the lipid droplet in the subintestinal vitelline vein [8, 38]. However, the first circulating cells are detected the 7th dpf. The ventricle and the atrium are separated by thicker walls and the atrio-ventricular valve localized between them (Fig. 5d). In the meantime, pectoral buds have evolved into pectoral fins and are clearly visible on living embryo (Fig. 5a, e, g). The tegument of the fins contains club and mucous cells. Moreover, the renal system is composed of pronephric ducts and urinary bladder after the end of intestine. The pronephric ducts are localized at each side of the myotomes (Fig. 5e) and organized in one layer of cuboidal cells forming a tube. The myotome and the chord are already well differentiated (Fig. 5f). From 7 dpf, first organs associated with the digestive system begin to differentiate as described below. In the same manner the eyes begin to be well formed (Fig. 5c). Few embryos begin to hatch from 6 dpf in the crates of the hatchery. No hatching gland and thus hatching enzyme releasing was observed in this study.

#### Organogenesis 3 (O3) stage from the differentiation of the organs associated to the digestive system to the mouth opening (7–10 dpf)

**Fig. 6:**
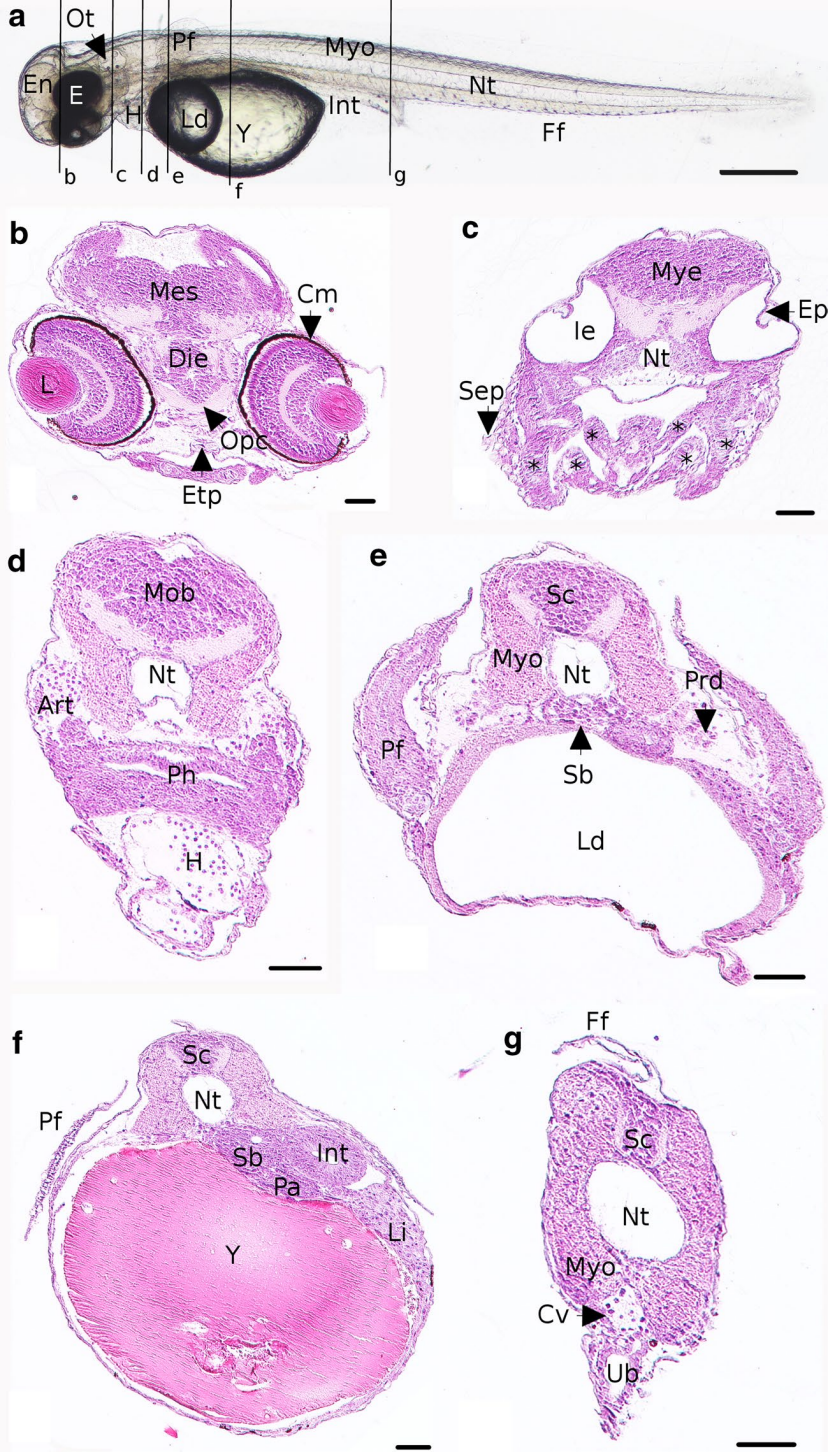
Detailed description of an embryo at 9 dpf representative of the end of the O3 period. **a** Whole embryo. *Black lines* represent the location of transverse sections shown in the following images. **b**–**g** Transverse sections. **b**, **c** Sections of the head. **d**–**f** Section of the trunk. **g** Section of the tail. *Art* artery, *C and stars *in **c** cartilage, *Cm* choroïdal melanocytes, *Cv* caudal vein, *Dien* diencephalon, *E* eyes, *En* encephalon, *Ep* epithelial projection, *Etp* ethmoïd plate, *Ff* finfold, *H* heart, *Ie* inner ear, *Int* intestine, *Ld* lipid droplet, *Li* liver, *Mes* mesencephalon, *Mob* *medulla oblongata*, *Mye* myelencephalon, *Myo* myotome, *Nt* notochord, *Opc* optic nerve chiasma, *Ot* otoliths, *Pa* pancreas, *Pf* pectoral fins, *Ph* pharynx, *Prd* pronephric duct, *Sb* swim bladder, *Sc* spinal chord, *Sep* squamous epithelium, *Ub* urinary bladder, *Y* yolk. *Scale bars* represent 500 µm for **a** 50 µm for **b**–**g**

Concerning the circulation system, large arteries are visible from the 8th dpf. They are located on each side of the somites, above the pharynx and more prominent at 9 dpf (Fig. 6d). The heart is composed of three well-developed chambers among which the *bulbus arteriosus* that appears at that time. The blood circulation makes sinuses in the yolk (Additional file 4) and leaves it toward the ventral part of the embryos. The heart beating is slow with a mean of 78 ± 0 beats per minute recorded (*n* = 3). The pigmentation appears in the ventral side of the embryos and spreads along the antero-posterior axis. In addition, at 9 dpf, one of the important events is the apparition of the cartilages (Fig. 6c). The first one to differentiate is the chondrocranium (cranial skeletal element) composed of the ethmoïd plate (Fig. 6b) and the parachordal which is paired on each side of the notochord. The second part of the cartilages structure is the pharyngeal cartilages which are divided into several elements: basihyal, basibranchial, hyosymplectic and palatoquadrate cartilages (Fig. 6c, black stars). Chondrocytes are also observed into pectoral fins (Fig. 6e). In the same time, in the inner ear, epithelial projections are visible (Fig. 6c). On histological sections, under the caudal vein, at the end of the rectum, the urinary bladder is apparent (Fig. 6g). The finfold surrounding living embryos (Fig. 6a) is also present above the spinal chord in a transverse section (Fig. 6g). The last stage of cartilage development is carried out the 10th dpf, with the formation of ceratobranchial arch and Meckel’s cartilage. The circulation system is developing with a visible cell circulation between the somites and toward gill arches on living embryos. At the end of the stage, the first peristaltic undulations occur. At the same time the mouth of the embryo opens. The main hatching period begins from 9.5 dpf but most of this period lasts over O4 and O5 stages. As a whole, we observed that hatching does not correspond to a specific stage because it lasts for 5 days on average (from the first embryo’s hatching to the last one within one spawn). Moreover, the onset of this phase is different between spawns (6–14.5 dpf) with high rates of hatching between 9 and 14.5 dpf. Interestingly, the developmental timing is the same if the embryo is still in the chorion or in the free form (Fig. 10a, b).

#### Organogenesis 4 (O4) stage from the mouth opening to the first coordinated movements (10–11 dpf)

During this period, the peak of hatching begins and free embryos cannot swim continuously but move toward the surface with short times of uncontrolled movements of fins and times of passive sinking. Except for the gills differentiation, no evident morphological modifications occur during that period.

#### Organogenesis 5 (O5) stage from the first coordinated movements to the first food intake (11–15 dpf)

**Fig. 7:**
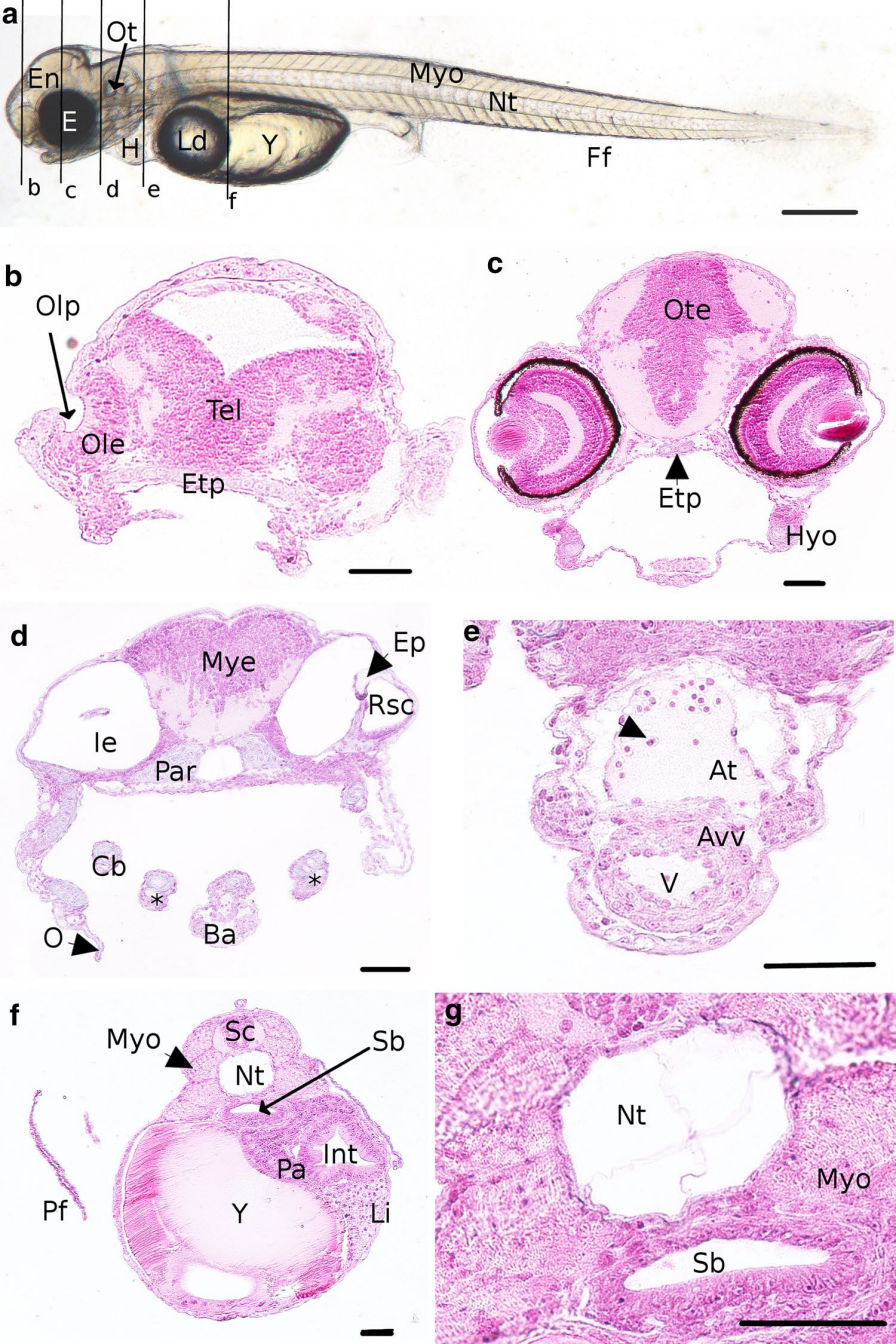
Detailed description of an embryo at 11 dpf representative of the O4 period. **a** Whole embryo. *Black lines* represent the location of transverse sections shown in **b**–**f**. **b**–**g** Transverse sections. **b–d** Sections of the head. **e** Section of the heart the *arrowhead* shows an erythrocyte. **f** Section of the trunk. **g** Detail of the section **f** showing the vacuolated notochord and the difference of epithelium thickness between the ventral and the dorsal sides of the swim bladder. *At* atrium, *Avv* atrioventricular valve, *Ba* *bulbus arteriosus*, *Cb and stars* ceratobranchial arch, *E* eyes, *En *Encephalon, *Ep* epithelial projection, *Etp* ethmoïd plate, *Ff* finfold, *H* heart, *Hyo *hyosymplectic, *Ie* inner ear, *Int *intestine, *Ld* lipid droplet, *Li* liver, *Myo* myotome, *Nt *notochord, *Ole *olfactory epithelium, *O* operculum, *Olp* olfactory pit, *Ot *otoliths, *Ote* optic tectum, *Pa* pancreas,* Par *parachordal, *Pf *pectoral fins, *Rsc *rostral semicircular canal, *Sb *Swim bladder, *Tel* telencephalon, *V* ventricle, *Y *yolk. *Scale bar* 500 µm for **a** and 50 µm for **b**–**g**

After the 11th dpf, the movements of the pectoral fins are more coordinated, allowing embryos to go toward the water surface. At the same moment, the gills anlage can be noted on living embryos. Below the hyosymplectic cartilage, opercula are developed and clearly observable. In the inner ear, epithelial projection forms the rostral semicircular canal of the vestibular system (Fig. 7d). At 11 dpf, embryos possess differentiated chambers that compose the heart (Fig. 7e), separated by the atrio-ventricular valve. The wall of the ventricle appears thicker than in the atrium. Blood cells (Fig. 7e, arrowhead) could be seen in the atrium. The heart beating is between 136 and 142 beats per minute (at 12 and 14 dpf, respectively). Melanophores line the ventral part of the embryos and a sporadic pigmentation is present toward the somites (Fig. 7a). On living embryos, first movements of the jaw are observed during the 12th dpf.

### First oral feeding (15 dpf)

Two independent experiments were conducted to determine the time of the first oral feeding. For each spawn (total number of spawn = 10), embryos were isolated from their hatching dates and alive *Artemia* nauplii were proposed as food daily. In every case, the moment of the first oral feeding appeared at 15th dpf in free embryo, independently of their hatching date. As this phenomenon always appears at a very specific moment, it corresponds to an important threshold for the embryo. Larvae still have a small yolk and a lipid droplet (Fig. 4e) indicating that they mainly entered a mixed feeding period.

**Fig. 8:**
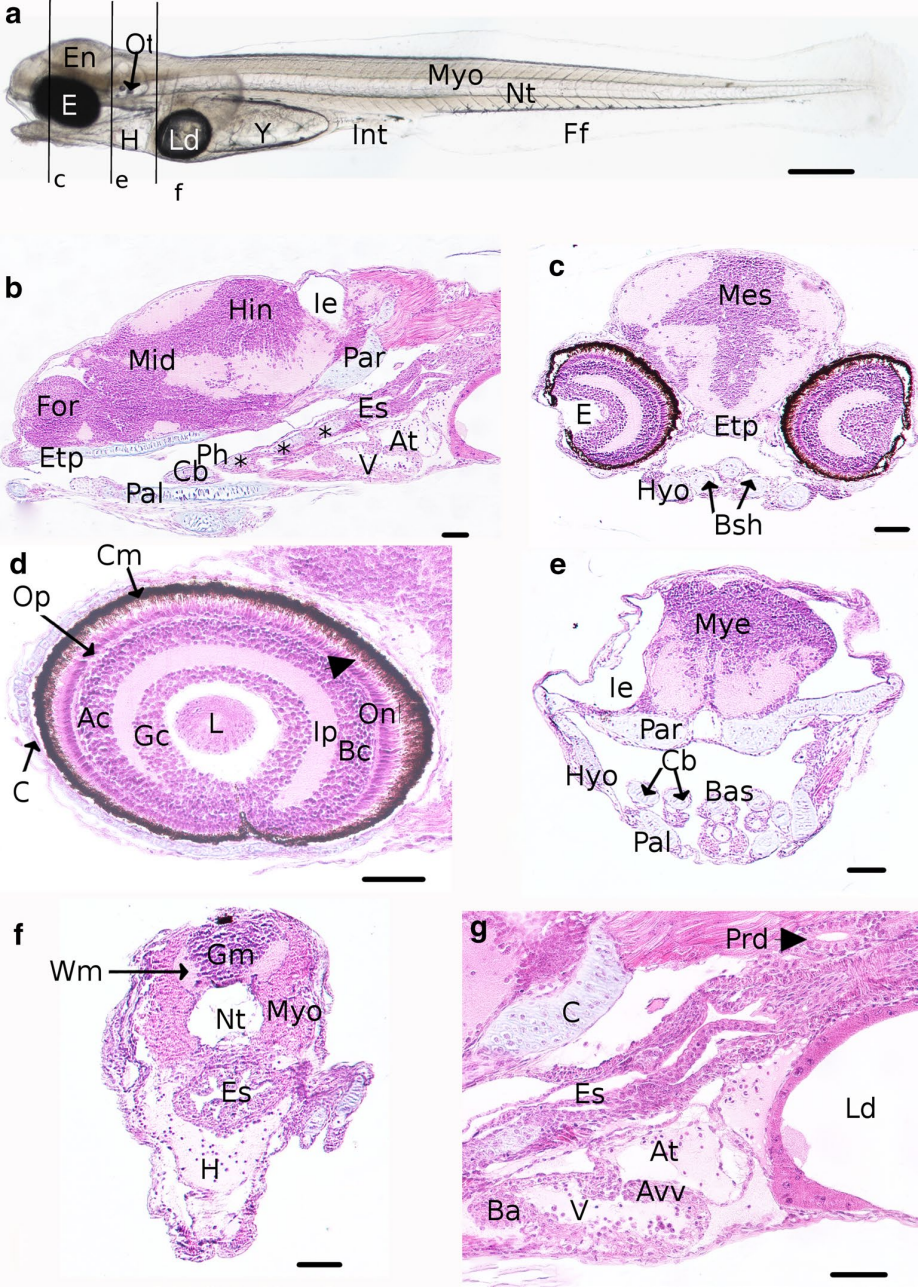
Detailed description of an embryo at 15 dpf at the end of the O5 period. **a** Whole embryo. *Black lines* represent the location of transverse sections shown in **c**, **e**, **f**. **b** Sagittal sections of the head. **c** Section of the head. **d** Sagittal section of the eye. The *arrowhead* shows the visual cells. **e** Section of basis of the head. **f** Section of the trunk. **g** Sagittal section of heart. *Ac* macrine cells, *At* atrium, *Avv* atrioventricular valve, *Ba* *bulbus arteriosus*, *Bas* basihyal, *Cb* *and stars* ceratobranchial arch, *Bc* bipolar cells, *C* cartilages, *Cm* choroïdal melanocytes, *E* eyes, *En* Encephalon, *Es* esophagus, *Etp* ethmoïd plate, *Ff* finfold, *For* forebrain, *Gm* gray matter, *H* heart, *Hin* hindbrain, *Hyo* hyosymplectic, *Ie* inner ear, *Int* intestine, *Ip* inner plexiform, *L* lens, *Ld* lipid droplet, *Mes* mesencephalon, *Mid* midbrain, *Myo* myotome, *Nt* notochord, *Onl* outer nuclear layer, *Op* outer plexiform, *Ot* otoliths, *Pal* palatoquadrate, *Par* parachordal, *Ph* pharynx, *Prd* pronephric duct, *V* ventricle, *Wm* white matter, *Y* yolk. *Scale bars* represent 500 µm for **a** and 50 µm for **b–g**

**Fig. 9:**
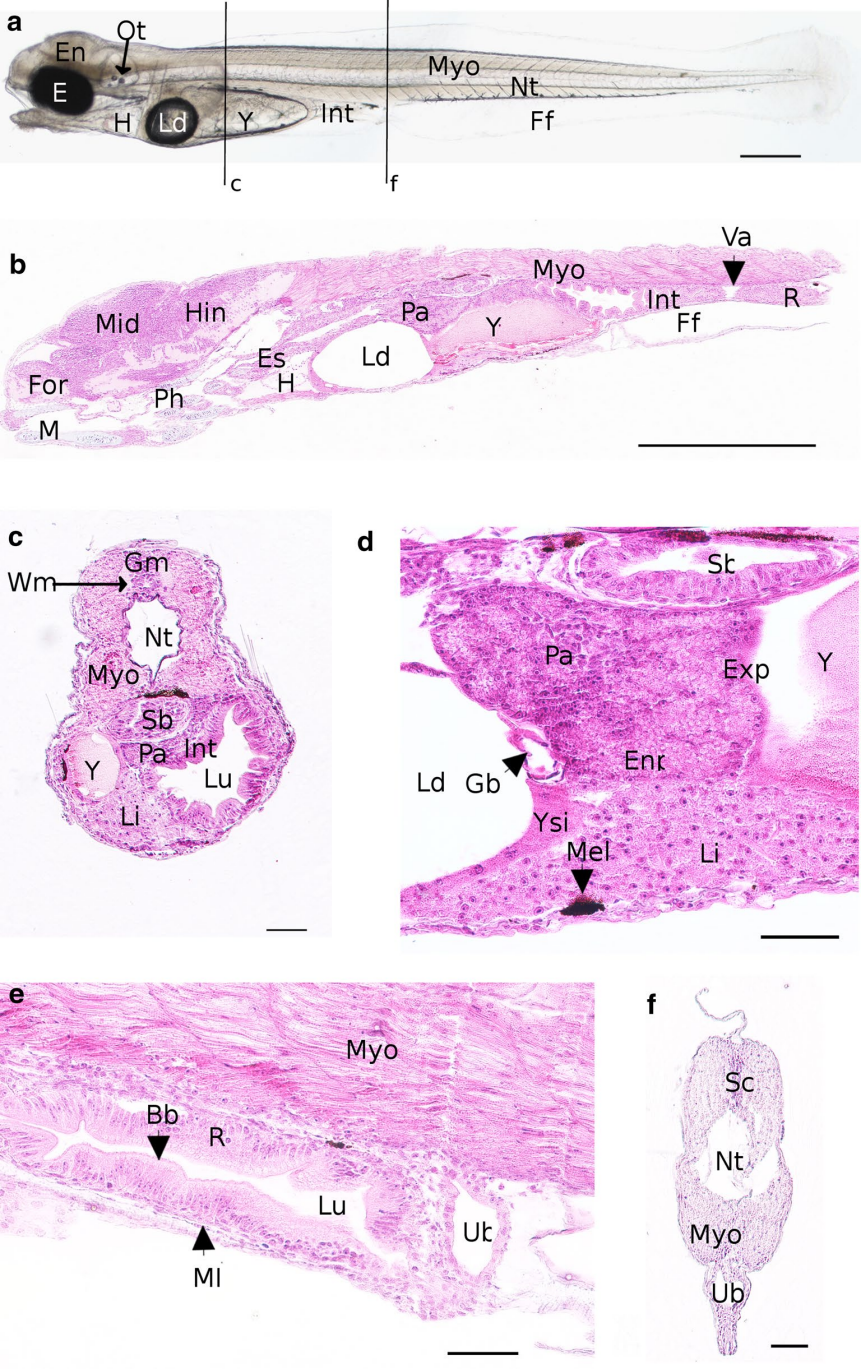
Detailed description of an embryo at 15 dpf at the end of the O5 period. **a** Whole embryo. *Black lines* represent the location of transverse sections shown in **c** and **f**. **b** Sagittal section of the anterior part of the embryo. **C** Section of the trunk. **d** Glands belonging to the digestive system. **e** Sagittal section of rectum. **f** Transverse section of the tail. *Bb* brush border, *E* eyes, *En* encephalon, *Enp* endocrine part of the pancreas, *Exp* exocrine part of the pancreas, *Ff* finfold, *For* forebrain, *Gb* gall bladder, *Gm* gray matter, *H* heart, *Hin* hindbrain, *Int* intestine, *Ld* lipid droplet, *Li* liver, *Lu* lumen, *M* mouth, *Mel* melanophores, *Mid* midbrain, *Ml* muscle layer, *Myo* myotome, *Nt* notochord, *Ot* otoliths, *Pa* pancreas, *R* rectum, *Sb* swim bladder, *Ub* urinary bladder, *Va* valve, *Wm* white matter, *Y* yolk, *YSL* yolk syncytium layer. *Scale bars* represent 500 µm for **a** and 50 µm for **b–f**

The anatomy of the young larva shows that cartilages are composed of chondrocytes and appear clearly differentiated from the other tissues and colored in blue on histological sections (Fig. 8b, c). These cartilages are divided in two main parts: the chondrocranium comprising the cranial skeletal elements, and the pharyngeal cartilages (Fig. 8c, e). It forms the musculoskeletal system with the myotomes (mainly future striated muscles), the notochord (supporting axis of the embryo) and the pectoral fins (Fig. 8a). A part of the circulation system is illustrated in the Fig. 8g with the heart composed by the *bulbus arteriosus*, the ventricle, the atrio-ventricular valve and the atrium. The blood flow will pass through the atrium, the ventricle pumping it into the *bulbus arteriosus*. On living embryos, melanophores line in the ventral side of the embryo, along the myotomes and on the yolk (Fig. 9a, d). The pigmentation is also present over the swim bladder (Fig. 9c) and on the rectum (Fig. 9e). The finfold starts to differentiate, forming lobes at the localization of the future caudal fin.

### Comparison of the developmental process between hatched embryos and their unhatched counterparts

**Fig. 10:**
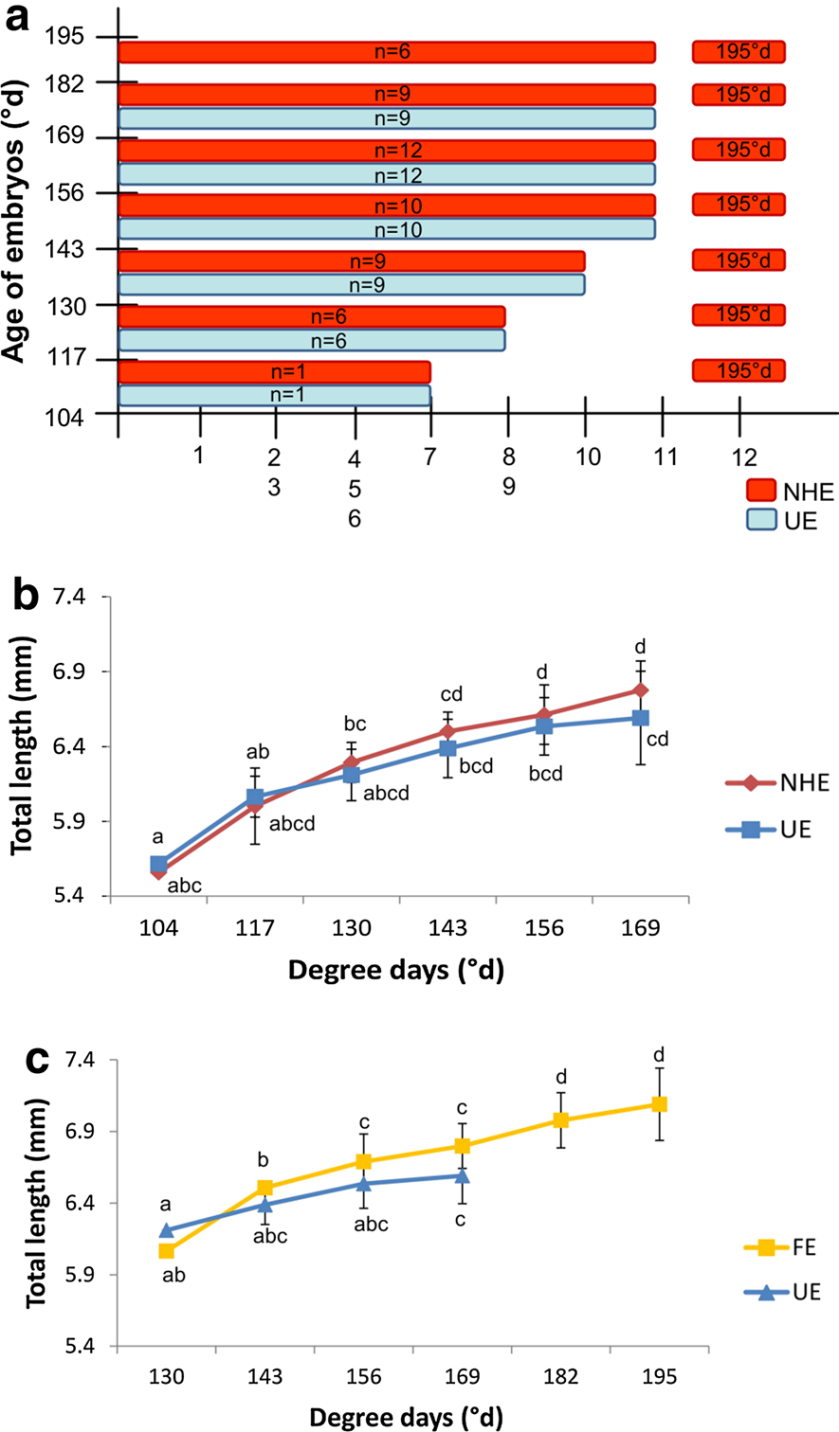
Comparison of the developmental process between free and embedded embryos. **a** Comparison of the developmental progress of newly hatched embryos (NHE) and unhatched embryos that still remain in the envelope (UE) at the same age. Twelve criteria were evaluated at the date of hatching. Observations have been done on embryos from four spawn, *n* correspond to the total number of embryos that hatched at a particular date and were used for this study. One embryo from one spawn begun to hatch at 8 dpf, while the others begun at 9 or at 11 dpf. Numbers correspond to the criteria as follows: *1* circulation in the yolk, *2* embryo straightening, *3* urinary bladder, *4* eye pigmentation, *5* ventral circulation, *6* bile, *7* melanophores on the body, *8* peristaltic undulations, *9* mouth open, *10* coordinated pectoral fins movement, *11* jaw movements and *12* first oral feeding. **b** The total length of newly hatched embryos (NHE) were measured and compared to the ones of embryos of the same age that remained in the envelope (UE) (*n* = 3 group UE or NHE/date of hatching/spawn, three spawn were used). Data correspond to the mean value of the three different spawns ± SD. Significant differences were observed according to the hatching date (*different letters*) but not the status hatched or unhatched embryos *p* < 0.05. **c** From the day after hatching, embryos were considered as free embryos (FE) their total length was measured daily and compared to unhatched embryos (UE) of the same age. (*n* = 3 group UE or FE/date of hatching/spawn, three spawn were used). Data correspond to the mean value of the three different spawns ± SD. Significant differences were observed according to the hatching date (*different letters*) but not the status hatched or unhatched embryos *p* < 0.05

In the present study, we observed that the hatching period elapses for several days while the first oral feeding took place at one particular moment (15 dpf) in every spawn. In order to determine whether there was a difference of development between newly hatched embryos and their not yet hatched counterparts we performed a comparison on four independent spawn. The hatching period lasts for 5 days (65°d) for each spawn. Each day of hatching, embryos that newly hatched (NHE) were sampled and observed to determine their stage of development. This stage was compared to the unhatched embryos (UE) of the same spawn (Fig. 10a). It appeared that embryos that hatched naturally didn’t present any difference in term of progression in the embryogenesis compared to their unhatched counterparts at the same age. Moreover, their mean size was not significantly different from unhatched embryos (Fig. 10b). Interestingly, this experiment shows that at the spawn level, the hatching period can last for several days and that embryos can hatch with various abilities. These data strongly support that embryos can reach various stages before their moment of hatching. In addition, in this experiment again, all embryos begun to eat at 15 dpf (195°d), whatever their hatching date. In these conditions, the first oral feeding may rather represents the embryonic-to-larval transition because they always occur at the same developmental stage.

To go further, we measured the size of each naturally free embryo (FE) from their hatching to the first oral feeding. We compared these sizes to those of embryos of the same age and the same spawn that were removed from gangue with forceps (UE) (Fig. 10c). Data show no significant differences between FE and UE embryos at a particular date. The only significant difference was obtained between sizes of embryos at various ages, showing a similar growth whatever if they hatched or remained in the gangue. These data suggest that there is no difference in the growth rate depending on living environment (still in the egg or free in the water).

### Description of the eyes development

The eyes are the first visible sign of cellular differentiation and develop from O1 to O3 stages. First, the optic capsules develop symmetrically on the embryo’s head (Fig. 3j) and soon after, the crystalline lenses appear from the 4th dpf (Fig. 4a) in the same time than the marginal zone. From 5 dpf, the outer nuclear layer appears in the optic capsule. Five different cell layers are observed from day six post-fertilization as illustrated on histological section of 7 dpf embryos (Fig. 5c). From this stage, the ganglion cells layer is the most inner layer of the eyes and the inner plexiform contains several rounded cells sibling amacrine and ganglion cells. The two cell types of the inner nuclear layer, amacrine and bipolar cells, are not yet differentiated while the visual cell layer is a light staining and composed of photoreceptor precursors. The outer nuclear layer consists of a row of dark-staining columnar cells (Fig. 5c). The 7th dpf, the eye’s pigmentation begins with a thin layer of black cells on the outer of optic cups (Fig. 5c, arrowhead). This pigmentation is not clearly visible on all histological sections or in living embryos. The optic nerves are developed at the same stage (Fig. 5c) and form a chiasm between the optic cups, under the hypothalamic, part of the diencephalon and more visible at 9 dpf (Fig. 6b).

From 8 dpf, the eyes diameter measures around 312 ± 16 µm (*n* = 16) and a new external layer of cells appears definitively in the eyes: the retinal pigmented epithelium with choroïdal melanocytes that lend a black pigmentation to the eyes (Fig. 6b). This dark pigmentation is visible on living embryos macroscopically (Fig. 4c) and marks the beginning of the eyed stage. Moreover, the outer plexiform layer forms a thin cover between the outer nuclear layer and the bipolar cells. At high magnification, on 11 dpf histological sections, cones and outer segment of cones are observed in visual cell layer of eyes. From 15 dpf, the structure of the retina is clearly discernible (Fig. 8d). In addition, a layer of chondrocytes forms a ring around each eye (Fig. 8d).

### Description of the digestive system ontogeny

The digestive tube is first visible from 4 dpf. It takes place dorsally to the yolk with a simple digestive tube composed of an undifferentiated epithelium. This intestine anlage already exhibits a lumen. From the 7th dpf, several organs associated with the digestive system begin to appear as the liver and the pancreas, which are close to the intestine (Fig. 5g). This differentiation marks the O2 to O3 transition. In the meantime, the esophagus also differentiates. The digestive tract is now a long tube localized in the left side of embryo. Its epithelium is composed of a monolayer of tall and aligned thin enterocytes with brush border at the apex (Fig. 9e). The pharynx and the gall bladder appear at the 8th dpf even if the gall bladder becomes more obvious at the end of embryonic period, between the lipid droplet and the pancreas, with a very thin wall (Fig. 9d). In the meantime, a yellow substance begins to be secreted and leaks in the posterior intestine until 11 dpf.

At 9 dpf, the swim bladder can be observed on histological sections, just below the notochord (Fig. 6e, f) and near the intestine. It is isolated from the yolk and consists of an oval structure composed of cuboids cells lining a central lumen. At the same stage, the diameter of the digestive tract varies along the antero-posterior axis and is thinner in the anterior and posterior regions than in the median region.

**Fig. 11:**
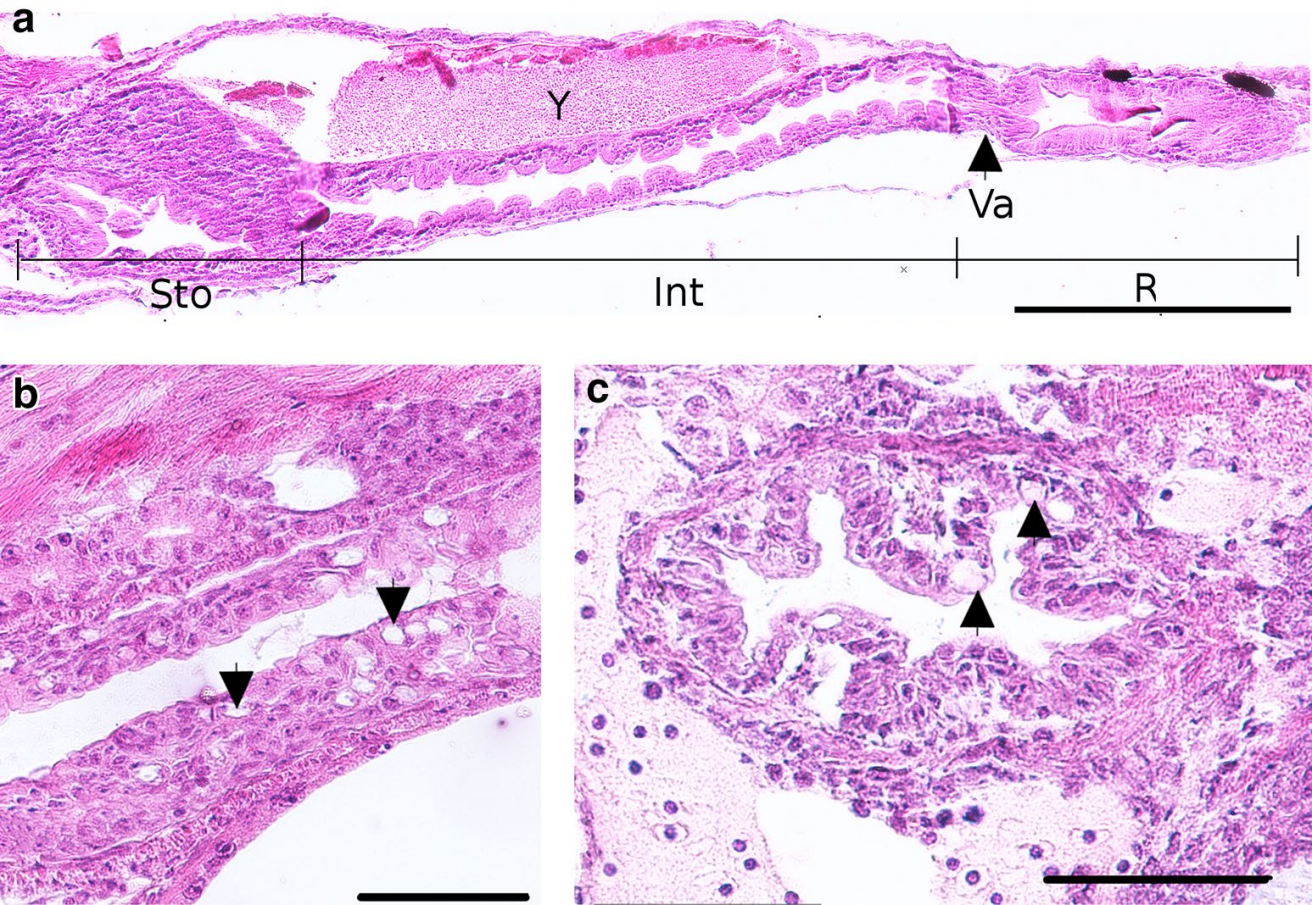
Detailed description of the digestive system at 15 dpf. **a** Coronal section of the trunk presenting all the gut. *Sto* stomach, *Int* intestine, *R* rectum, *Va* ileorectal valve, *Y* yolk. **b** Detail of the esophagus with globlet cells (*arrowheads*). **c** Transverse section of esophagus with goblet cells (*arrowhead*) and folds. *Scale bars* represent 50 µm

First peristaltic movements are visible at 10 dpf in the posterior part of the intestine whereas the same phenomenon is observed in the anterior part of the intestine at 14 dpf. In the same time, the mouth opens (Fig. 4d). It marks the transition from O3 to O4 stages. At 11 dpf, the liver is composed of hepatocytes with large central nuclei and the intestine mucosa appears folded, in order to increase its surface area (Fig. 7f). All the organs involved in the digestive function take place on the left side of embryo. For the first time in the embryogenesis, a new organization appears in esophagus with many epithelial folds and the differentiation of goblet cells (Fig. 11b). The swim bladder presents various cells types: on the dorsal side, small cells forming the wall of the oval structure and on the ventral side, tall cells that confer a large aspect of the wall (Fig. 7g). It is surrounded by mesenchymal cells forming a fibrous lining (also described by Chevey [32]). At that stage, the anus opens.

A dark pigmentation begins to develop on the dorsal part of the swim bladder at 12 dpf, and is easily observable at 15th dpf on living embryos (Fig. 9a) and histological sections (Fig. 9c, d). The esophagus is situated above the heart and contains many folds in the mucosa and the goblet cells (Figs. 8f, 10b, c). The digestive tract is divided into three main parts: (i) the future stomach separated from the intestine by a sphincter, (ii) the anterior intestine and (iii) the posterior intestine also called rectum. This subdivision is observable on coronal or sagittal sections as in the Fig. 11a. The anterior and posterior segments of the intestine have different lengths (the first part is longer than the second one, 596.1 ± 20.2 and 244.7 ± 14.2 µm, respectively) and are separated by a constriction called ileorectal valve (Fig. 9b). Anterior and posterior intestines have different morphological organizations which could be explained by folds in the wall (Fig. 11a). Indeed, in several teleost species, the anterior region of gastrointestinal tract possesses primary and secondary folds (Fig. 9c) whereas the rectum presents a change on mucosal folding pattern. The epithelial layer of the gut is composed of thin and elongated enterocytes with an absorptive function, surrounded by a muscular layer and, in the center of the tract, the lumen (Fig. 9e). The brush border of the enterocytes increases again the intestinal absorption surface. At 15 dpf, the digestive glands are fully differentiated. The pancreas (Fig. 9d) includes two distinct glandular systems (endocrine and exocrine), and the liver is positioned ventrally to the pancreas and closely linked to the yolk. Between these two glands, the gall bladder is observed (Fig. 9d).

## Discussion

### Establishment of the developmental table of the Eurasian perch

**Fig. 12:**
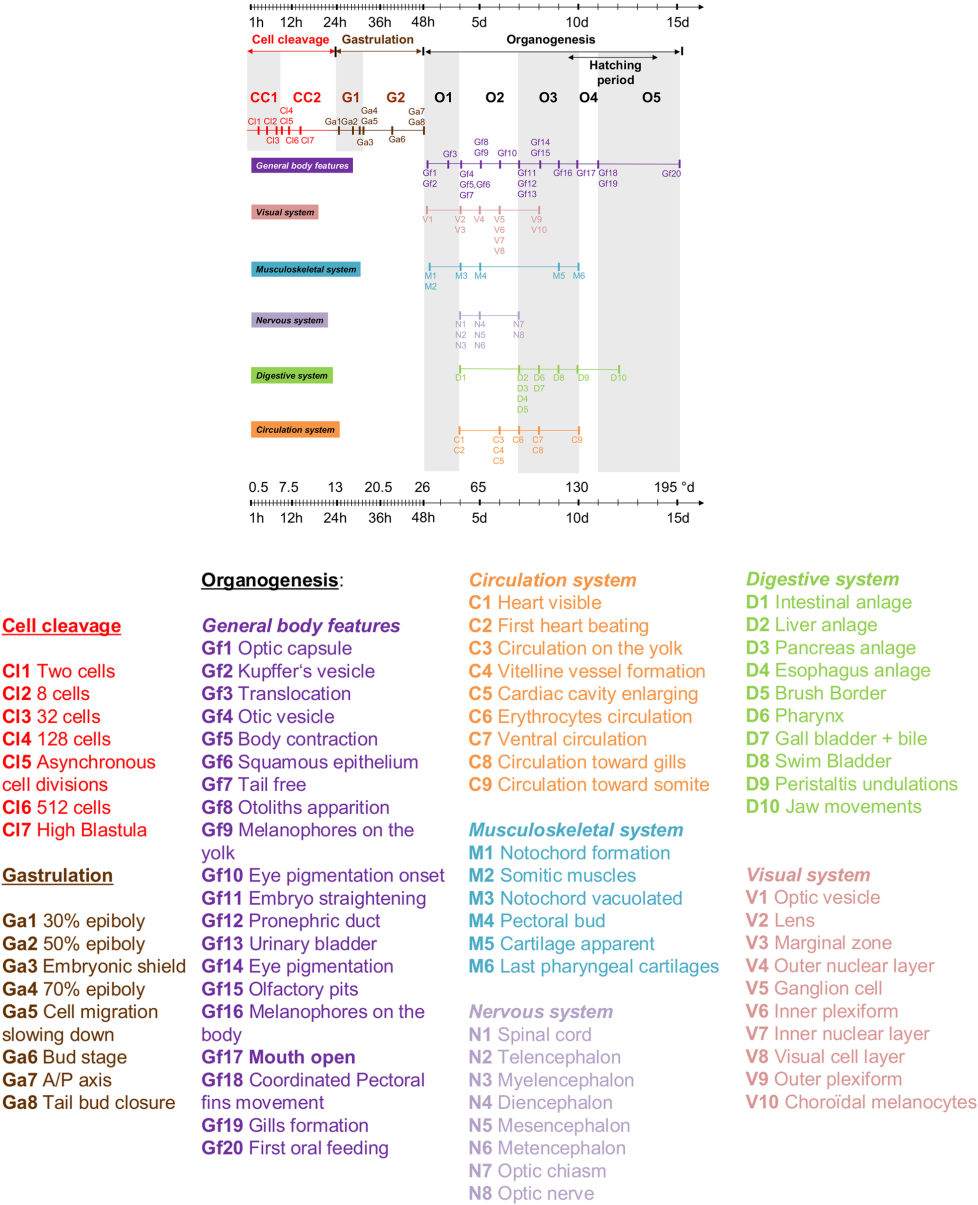
Developmental table of the Eurasian Perch (*P. fluviatilis*). The time scale is given in hours (h) and degree days (°d). First a general table of embryonic stages as described in the present papers. Then, the first observation of an organ implementation or a new ability was highlighted on living embryos or histological studies. Observations were classified according to their involvement in the visual, musculoskeletal, nervous, digestive and circulation systems. The general body features characterize observation performed in living embryos and that could be easily noticed to distinguish developmental stages

The present work first aims at describing accurately the Eurasian perch development. Data show that its timing can present variations preventing to follow a strict developmental table as those proposed for fish model species [1, 5, 6]. Moreover, the hatching period elapsed for several days and individuals did not hatch at the same developmental stage while the first oral feeding occurred at the same time for every embryo. In that context, we choose to determine an alternative method to describe perch development that is flexible enough to take into account this variability while being rather precise to describe accurately the development. Descriptions of steps and thresholds as defined by Balon [9] allow high flexibility but lack sometimes accuracy, especially during the early stages. We, thus, decided to use this model to describe perch development while defining more accurate stages in the beginning of the embryogenesis. First periods have been defined according to the cellular status (division, migration or differentiation). In a second time, thresholds were established thanks to the acquisition of new abilities, defining in the mean time steps that were carefully described. Some thresholds were established according to previous work on other percids [8, 39–46], while others were defined to improve the developmental table and thus add accuracy to the description. Such developmental table not only allows an easier comparison between individuals within a species, but also, between fish species by translating the time scale into a degree day scale. The main description of perch embryogenesis with the definition of steps and an accurate description of the first observation of each organ is summarized in the Fig. 12. As a whole, the total embryogenesis duration elapses for 15 days at 13 °C (195°d) from the fertilization to the first oral feeding. It is comparable to the yellow perch (185.6°d [48]). Other percids exhibit shorter or longer development from activation to the first oral feeding (Table 3).

**Table 3:** Comparison of the developmental of the Eurasian perch with other percid fish

Scientific name	Common name	Adult size (cm)	Spawning substratum	Incubation temperature (°C)	Reproductive season	Egg size (mm)	Total embryonic duration	CC1 onset	CC2 onset	G1 onset	G2 onset	O1 onset	O2 onset	O3 onset	O4 onset	O5 onset	Size at hatching (mm)	Hatching duration	Larval size (first feeding) (mm)	References
*Etheostoma*																				
*Etheostoma caeruleum*	Rainbow darter	5.3	Burried in the substrat	20	Spring	1.9–2.2	243.9	1.4	nd	17.4	nd	25.1	43.4	85	120	180	8	20	9.6	[46]
*Gymnocephalus*																				
*Gymnocephalus cernuus*	Ruffe	12	Demersal	16–23	Early summer	1	154.1	3.4	nd	nd	nd	18.7	35.7	80.9	134.6	nd	3.5–3.8	42.7	4.4–4.9	[41, 47]
*Perca*																				
*Perca fluviatilis*	Eurasian perch	25	Fixed on plants or stones	13	Early spring	1.75	195	1.4	3.9	13	16.9	27.1	52	91	130	143	5	65	6	This work
*Percina*																				
*Percina caprodes*	Logperch	12.5	Burried in the substrat	20	Late spring	1.23	164.4	1.3	nd	nd	nd	19.9	41.5	51.5	156	nd	6.2	10.6	6.2	[39, 45]
*Sander*																				
*Stizostedion vitreum*	Walleye	54	Demersal	15	Early spring	1.85	236.3	1.6	nd	16.4	nd	28.9	52	nd	153.6	nd	6.8–7.3	67.5	9–9.7	[8, 47]
*Zingel*																				
*Zingel streber*	Danube streber	12	Burried in the substrat	12–17	Early spring	1.94	360	2.2	nd	7.7	nd	22.5	82.5	128.5	210	240	6.4–7.6	75	9.2	[44]

Each fish species represent each family of percid. They were chosen because the description of their development is currently the most accurate among their family. Intervals onsets or durations are given in degree days (number of days × temperature)

### The zygote period

The zygote step corresponds to the egg hardening and the formation of the perivitelline space and the blastodisc. The lipid reserves are separated into two independent parts, the yolk and the lipid droplet also called oil globule as described for all percid species and some other non percid fish. But the presence of this droplet is not general for every fish species. It has been shown in the walleye that both compartments are composed of different lipids. Indeed the yolk is mainly composed of lipoproteins and polar lipids (e.g., phosphatidylcholine and phosphatidylethanolamine), the oil globule is composed by neutral lipids, mainly triacylglycerols (TAG) [49]. Moreover, Wiegand et al. [50] noticed that the yolk is used predominantly during early embryogenesis while the lipid reserves in the droplet are used later during the embryogenesis.

### The cell cleavage period

After 2.5 h (1.4°d), the cell cleavage period begins. This step never occurs when ova were not incubated with sperm and thus not fertilized (data not shown) on the contrary to other species as the herring or the stickleback for which unfertilized eggs can undergo cell cleavage [51]. The total duration of both zygote and cell cleavage periods are comparable to the rainbow darter and the walleye [8, 46] (Table 3) and last for about 24 h (13°d) for the perch. The control of the cell cleavage doesn’t seem to be only dependent upon the water temperature as the *Zingel streber* cell cleavage lasts for 12 h at a mean temperature of 15 °C (7.7°d) [44] which is in the same range of temperature than the one of the Eurasian perch. In every case, the cleavage is meroblastic due to a large yolk quantity, which remains uncleaved [52] and begins with a bilateral arrangement of the blastomeres with a regular cleavage pattern, as in other fish species [1–3, 6]. Nevertheless, after several divisions irregularities always occur in the division planes but the onset of this phenomenon was different for every species. In the present study, we noticed it at the 32 cells stage as for the sea bass and the medaka [1, 3]. In contrast, Chevey [32] reported it from 16 cells stage in *P. fluviatilis* as it is the case for the walleye [8] and it was even earlier for the logperch (eight cells) [45]. These irregularities could precede defects in the synchronization of the cell divisions as reported in zebrafish, for which both process appears from 128 cells stage [6]. The transition between synchronous and asynchronous cell divisions could correspond to the mid-blastula transition (MBT) which is characterized by the loss of synchronous cell division, cell motility, lengthening of cell cycles and the onset of zygotic transcription [53, 54]. In the present study, the cell division asynchrony also begins from 128 blastomere stage. We propose to subdivide the cleavage cell period into two steps, (CC1 synchronous cell division and CC2 asynchronous cell divisions). This subdivision could not be properly established in other percids developmental tables because the description of cell cleavage was not accurate enough (Table 3).

### The gastrulation period

The gastrulation period starts the second day post-fertilization with the first cell movements along the yolk, and lasts for about 26 h at 13 ± 0.5 °C unlike to Chevey [32] who described the gastrulation duration of 55–60 h. This huge difference is surprising and we do not have any plausible explanation. Nevertheless, our data were reproducible in every spawn whatever the spawning season and the origin of breeders and are related to those obtained with other percids (Table 3) suggesting that we may probably be closer to the reality. After the cell cleavage, several cell layers could be seen among which the yolk syncytial layer (YSL) situated between the yolk and blastomeres and characterized by a multinucleate layer, continuous with the yolk cytoplasmic layer. We observed it until 15 dpf indicating that it may have a role in the nutrient absorption all along the embryonic development. In addition, the YSL plays an active role during gastrulation as it gives most of the contractile force during the epiboly [52]. The embryonic shield is visible from 50 % epiboly as described in medaka and zebrafish [1, 6]. In *Etheostoma* species belonging to the percids, the germ ring and the embryonic shield formation occurs early at 25 % of epiboly [55]. Moreover, this timing seems to be crucial because premature or delayed involution may lead to embryonic lethality [55]. Kimmel et al. [6] noticed a pause during the zebrafish development. In our study, this arrest is not observed but a change in the velocity of the cellular front advancement can be observed from 70 % epiboly with deceleration of the blastoderm margin progression. This delay could be linked to the oil droplet covering by the embryo. Mc Elmann and Balon [8] have described in the walleye a progressive rotation of the embryos while the cellular layer was covering the oil droplet. This phenomenon occurs potentially because of a variation of the cell velocity between the ventral and the dorsal part of the embryo as observed in the zebrafish [6]. This rotation was described not only for the walleye but also in the northern logperch [45] but not in darters [46, 55] or in the *Gymnocephalus* species [43]. In the present study, in most cases we didn’t observe such rotation (only once among the embryos we observed). We rather observed a very fast covering of the oil droplet as if tensions were accumulated before this covering and released quickly (Additional file 3). This tension may be due to the deceleration previously described. We propose to subdivide the gastrulation thanks to cell velocity (G1 as the rapid cell movements and G2 from the deceleration).

### The organogenesis period

The boundaries between the gastrulation and the organogenesis are not always clear because organogenesis can be triggered before the tailbud closure as it is the case for *Perca fluviatilis* (Fig. 12) or other percid fish species as *E. caeruleum* [46]. In addition, it is clear that organogenesis will continue at least until the juvenile stage. We nevertheless choose to name the next period organogenesis in order to make the occasion to describe the beginning of the cell differentiation. The definition of thresholds took into account the acquisition of abilities for several functions/systems as the locomotion system (including fins, muscles and skeleton), the nervous system (central and peripheral including sensory organs), the circulation system (heart and vessels), the feeding/excreting system (including all organs associated to the digestive function and the kidneys), the respiratory system (mainly gills). Naturally, other systems as for example, the reproductive or the immune systems are important for the individuals but either their development will occur later (reproduction) or we did not identify any specific organs (e.g., spleen) with the techniques used in this work. As a whole, the organogenesis begins with the optic vesicle apparition 2 dpf (27.1°d). During this phase, the embryonic size varies from 5.32 mm ± 0.23 to 6.28 mm ± 0.22 as previously observed in *Perca fluviatilis* and *Perca flavescens* [32, 48].

One of the first organs to be visible is the transient Kupffer’s vesicle. It was described in 1868, and appears as a conserved structure among teleost fishes. This spherical and ciliated organ is localized in the posterior part of the embryo ventrally to the notochord. It plays a role in left–right development of the brain, heart and gut in Zebrafish embryo [56]. In addition, Kimmel et al. [6] suggest that cells lining the Kupffer’s vesicle may be responsible of formation of tail mesodermal derivatives as the notochord and muscles. Other tissues as somites, the central nervous system or the notochord then progressively appear (Fig. 12). During the process, it has been reported in the walleye, [8], that the embryonic body rotate around the yolk to move the head closer to the oil droplet. They called this movement the translocation. In the present study, we also observed a translocation of the embryo. Up to now it corresponds to the second observation among percids. At 4 dpf, the heart is visible and begins to beat. However, no liquid was already circulating. Burggren [57] suggested that the heart begins to beat to improve its morphogenesis, for convective blood flow and angiogenesis whereas nutrients and respiratory gases could be delivered by passive diffusion during the early life of the embryos. In addition, these first beatings are concomitant with the first spontaneous skeletal muscle contractions. It suggests that the smooth muscle cells of the heart and striated muscle cells of the somites undergo parallel development even if the heart differentiation is not distinguishable before. We propose that this step corresponds to a threshold according the saltatory theory. We then call this first step from the eye differentiation and first muscle contraction (body and heart) O1 for organogenesis 1.

A new step that we called O2 then begins until 7 dpf when organs associated to the digestive system appear. During that stage, the eyes are almost fully developed and several layers are distinguishable. A cartilage and an epithelium form the cornea and give a mechanical protection to the eyes. Just behind this cell layer the visual cells composed by photoreceptors take place. These cells are involved in the light perception and transmission. It exists two categories of photoreceptors, the cones enabling the fish to improve their perception of the preys and the rods, appearing later, and rather involved in light sensitivity of the fish (e.g., perception of a shadow coming from the surface) [58]. The full development of these cells usually allows the embryo to obtain their full visual acuity [58]. They form synapses with interneurons in the outer plexiform and their nuclei compose the outer nuclear layer. However, in the perch the outer plexiform layer is not yet visible suggesting that the neuronal connections are not yet possible and that the embryo is still blind. Interneurons are bipolar cells that make the link between photoreceptors and the ganglion cells. There nuclei take place in the inner nuclear layer and they form synapses with the ganglion cells in the inner plexiform [59]. Amacrine cells appear in the same layers and participate to the signal processing of the visual stimuli at the level of the bipolar/ganglion cells synapses. The ganglion cells allow the connection between retina and optic tectum belonging to the mesencephalon [59]. The retina corresponds to the whole cell layers from the melanocytes to the ganglion cells. From 7 dpf, the optic nerve forms a chiasm between the optic cups and continue into the opposite brain hemisphere [59]. Other organs further develop during the O2 step as the circulation system with the liquid circulation that begins in the vessels 2 days later (6 dpf). Several sinuses progressively appear on the yolk (Additional file 4), suggesting that this liquid mainly transport nutrients in the embryonic body.

The O3 period begins 7 dpf until the first peristaltic undulations are visible and mouth opens at 10 dpf. Most of the organs involved in the digestion function (pancreas, liver), in the excretion system (kidney, urinary bladder) and the intestine differentiation present a synchronous development during that step. The digestive tract of teleosts is not open on the yolk [51]. Instead, the yolk nutrients absorption is rather mediated through the blood circulation. The liver seems to play a particular role as it takes place close to the yolk. Chevey [32] observed that it develops a particular hepato-vitellin connection that may have a role in yolk sac resorption. Indeed, blood vessels coming from the yolk pass through the liver and by this way, deliver to the embryo the vitelline reserves absorbed by this gland. This close association between the liver and the yolk is described in several teleosts but not in *Danio rerio* [6]. From 8 dpf, the bile seems to escape from the digestive tract until 11 dpf. Similar descriptions have been made in the walleye and the salmon [8, 60]. The swim bladder differentiates from the digestive system during that step. No swim bladder inflation was observed in the present study. This phenomenon may probably occur later during larval stages. At 10 dpf, the mouth opens and first peristaltic undulations occur suggesting that the digestive system is morphologically ready. In the meantime, the gills fully develop allowing the embryos to breathe and thus acquire higher oxygen supply. The eye is fully pigmented at 8 dpf just before the peak of the hatching period. In the meantime, the outer plexiform is observed and thus animals probably begin to see. They mainly go toward the water surface to take air bubbles, required for swim bladder’s inflation or just by positive phototaxis of free embryos.

### Definition of the embryonic-to-larval transition

The question of the embryonic-to-larval transition is continuously discussed in the fish scientific community. Some authors argue that hatching corresponds to this transition because fishes develop new behavior at that stage while others prefer the first oral feeding [11–13]. Our study reveals that the hatching period onset occurred mainly from 9 to 14 dpf depending on the spawn, despite of parallel rearing in the same hatchery. Some spawn begun to hatch earlier (6 dpf) for no obvious reasons (breeders population, water quality…) because other spawn of breeders of the same population and incubated in the meantime in the same hatchery hatched between 9 and 14 days. It could be due to intrinsic genetic background of the breeders that was not tested. Interestingly, the hatching period elapses for 5 days (65°d) in average within a spawn. This is different from a previous study [29], describing hatching between the 14th and 15th dpf at 13.5 °C. Concerning other percid fish species, except the rainbow darter, the yellow pope and the logperch that hatch for few hours [7, 39, 42, 45], most of percid species exhibit a longer hatching period (Table 3). This long hatching period opened the question of whether embryos that hatch the first and the last day have reached the same developmental stage but with different speed or whether they hatch at several developmental stages. A first study described several hatching stages in *P. fluviatilis* depending on the embryonic length [29]. In the present study, we determined that embryos hatched at various developmental stages. Indeed, the following of 12 criteria allowed us to show that embryos that hatched 1 day reached a more advanced developmental stage than the ones that hatched the day before. This observation was not only done within a spawn but also between the four spawn that were used for this experiment. Moreover, as previously described by Trabelsi et al. [37], embryos that hatched at different dates had significantly different sizes. However, their size was not significantly different from their unhatched counterparts. As a consequence we can conclude that at the embryo level, hatching corresponds to an important threshold because they will need to adapt to a new environment. However, at the spawn level, hatching rather corresponds to a period during which embryos can free themselves from the eggs at various developmental stages. In addition, our data do not show any compensatory growth for the free embryos compared to their unhatched counterparts. Kestemont et al. [61] showed that *P. fluviatilis* embryos hatching earlier have more chances to survive than those that hatch later. This diversity of hatching dates may thus improve the survival success of the species in the wild. Such issue has not been addressed in the present paper. An inter-species comparison from the literature shows that *Gymnocephalus* sp., *S. vitreus* and *P. flavescens* hatch at different developmental stages [8, 43, 48]. Indeed, for *Gymnocephalus* sp. and *S. vitreus* young free embryos, the mouth is not yet well developed and not opened. This is not the case *for P. flavescens*. In addition, the eye pigmentation is absent for *Gymnocephalus* while they are well colored in *P. flavescens* and *S. vitreus*. These observations suggest that every species don’t need to reach the same level of abilities before leaving their envelope and survive in their environment. In addition, the hatching duration can be variable depending on the species (Table 3). No detailed study has been performed on other percid fish species to determine hatching stages. However, our data strongly suggest that it rather represents a step the limits of which vary between spawn within a species. In the meantime, the definition of a free embryo period also corresponds to a floating step the limit of which depends upon those of hatching. It would be interesting to perform such experiments in other fish species to better understand this important statement. In contrast to the hatching period, all embryos began to feed 15 days post-fertilization whatever their hatching dates. These data were observed in all tested spawn suggesting that they all reached a specific stage allowing them to feed. This narrow timing was quite surprising but it appears that for several fish species, the variation of developmental stage at the first food intake is weak compared to hatching that is more susceptible to external parameters [13]. One explanation could be that the most anterior part of the intestine does not undergo peristaltic movements before 14 dpf. So the food would stay blocked in the embryos and could lead to the individual death. A second explanation could be that the amplitude of the mouth opening is important enough to catch the prey before 15 dpf [62]. Finally, the last explanation could be that the digestive enzymes are not fully activated. Indeed, even if the digestive enzymes (trypsin, amylase, brush border alkaline phosphatase) are present in the pancreas before the mouth opening and present specific activities at hatching or few days later; their enzyme activity increases during the following days and reach a maximum at first oral feeding period (at least for trypsin) [63]. This increase of trypsin and amylase specific activity was also observed in the sea bass and appears directly linked to substrate’s concentration of ingested food [64]. All these data suggest that the first oral feeding rather corresponds to a threshold according to the saltatory theory and thus better define the embryonic-to-larval transition.

## Conclusion

Among the different lines of thoughts and definitions available to describe fish embryogenesis, we clearly believe that the saltatory theory proposed by EK Balon is more convenient although perfectible to describe fish development that presents timing variations. Indeed, taking into account the parallel development of several systems to acquire new abilities that are triggered promptly is wise. In addition, defining the first food intake also appears more precise to define the embryonic-to-larval transition than hatching that elapses for a long period within a spawn. However, a specific study of hatching should be performed to better understand the reasons of this huge difference of hatching dates not only within a spawn but also between spawns. On the contrary to EK Balon, and for the same reason (long hatching period), we did not take into account any free embryo period. We consider this “free embryo” period such as the hatching period as floating periods the limit of which can be flexible depending on intrinsic and extrinsic factors. We, thus, propose to describe perch development according to steps corresponding to cellular status and the acquisition of new abilities. This proposition could thus be used as canvas to describe the development of other non-model fish species and then allow intra-species and inter-species comparisons.

## Additional files

10.1186/s13227-015-0033-3 Time lapse video of the cell cleavage. Waving in the yolk are easily observable at each cell clevages. These waves were most important during the first cleavages. Four different sequences have been recorded. Cool water was added between each sequence to keep the water temperature at around 13°C. For the first sequence (from the beginning to 15 sec on the movie) 1 image was recorded every 2 minutes while for the 3 last sequences 1 image was recorded every 30 seconds. As a consequence, the first part of the movie has a speed of 6 images/sec and the last one 24 images/sec.
10.1186/s13227-015-0033-3 Time lapse video of the gastrulation. At 50% epiboly, the cell margin slows down before surrending suddenly the lipid droplet. Right after, the yolk rotates inside the embryo. Five sequences were recorded all at 1 image per minute. As a consequence the movie speed is 10 images/sec.
10.1186/s13227-015-0033-3 Real time record of the circulation on the yolk. Several sinuses are dug in the yolk at provide nutrients to the embryo. The speed corresponds to the real time and the movie speed is 15 images/seconds.
10.1186/s13227-015-0033-3 Real time record of the peristaltic undulations in the rectum. Smooth muscle contractions provoke the undulations of the posterior intestine. The speed corresponds to the real time and the movie speed is 14 images/seconds.

## Abbreviations

°C: celsius degree
°d: degree days
Ac: amacrine cells
ANOVA: analysis of variance
A: anterior
AP: animal pole
Art: artery
At: atrium
Avv: atrioventricular valve
Ba: bulbus arteriosus
Bas: basihyal
Bb: brush border
Bc: bipolar cells
Bl: blastoderm
C: cartilage
Cb: ceratobranchial arch
CC1 and CC2: cell cleavage steps 1 or 2
Cm: choroïdal melanocytes
Cv: caudal vein
D: dorsal
Dien: diencephalon
Dpf: days post fertilization
DV: dorso-ventral axis
E: eyes
EDF: extended depth of focus
En: encephalon
Enp: endocrine part of the pancreas
Ep: epithelial projection
Es: esophagus
Etp: ethmoïd plate
Exp: exocrine part of the pancreas
FE: free embryos
Ff: finfold
For: Forebrain
G1 and G2: gastrulation steps 1 or 2
Gb: gall bladder
Gc: Ganglion Cells
Gm: gray matter
H: heart
Hind: hindbrain
Hyo: hyosymplatic
Ie: inner ear
Inl: inner nuclear layer
Int: intestine
Ip: inner plexiform
hpf: hours post fertilization
L: lens
Ld: lipid droplet
Li: liver
Lu: lumen
M: mouth
mm: millimeter
Mel: melanophores
Mes: mesencephalon
Mid: midbrain
Ml: muscle layer
Mob: medulla oblongata
Mye: myelencephalon
Myo: myotomes
NHE: newly hatched embryos
Nt: notochord
O: operculum
O1 to O5: organogenesis steps 1–5
Ob: olfactory bulb
Oc: otic capsule
Ole: olfactory epithelium
Olp: olfactory pits
Onl: outer nuclear layer
Opc: optic nerve chiasma
Opn: optic nerve
Ote: optic tectum
P: posterior
Pa: pancreas
Pal: palaquadrate
Par: parachordal
Pf: pectoral fins
Prd: pronephric ducts
R: rectum
Rsc: rostral semicircular canal
Sb: swim bladder
Sc: spinal chord
SD: standard deviation
Seq: squamous epithelium
Sto: stomach
Tel: telencephalon
Ub: urinary bladder
UE: unhatched embryos
V: depending on the context ventral il the Fig. 3 or ventricle in the Figs. 5, 7 and 8
Va: depending on the context valve in the Fig. 9 or ileorectal valve in the Fig. 11
Vc: visual cells
VP: vegetative pole
Wm: white matter
Y: yolk
YSL: yolk syncitial layer
Cl1: two cells
Cl2: 8 cells
Cl3: 32 cells
Cl4: 128 cells
Cl5: asynchronous cell divisions
Cl6: 512 cells
Cl7: high blastula
Ga1: 30 % epiboly
Ga2: 50 % epiboly
Ga3: embryonic shield
Ga4: 70 % epiboly
Ga5: cell migration slowing down
Ga6: bud stage
Ga7: A/P axis
Ga8: tail bud closure
Gf1: optic capsule
Gf2: Kupffer‘s vesicle
Gf3: translocation
Gf4: otic vesicle
Gf5: body contraction
Gf6: squamous epithelium
Gf7: tail free
Gf8: otoliths apparition
Gf9: melanophores on the yolk
Gf10: eye pigmentation onset
Gf11: embryo straightening
Gf12: pronephric duct
Gf13: urinary bladder
Gf14: eye pigmentation
Gf15: olfactory pits
Gf16: melanophores on the body
Gf17: mouth open
Gf18: coordinated pectoral fins movement
Gf19: gills formation
Gf20: first oral feeding
C1: heart visible
C2: first heart beating
C3: circulation on the yolk
C4: vitelline vessel formation
C5: cardiac cavity enlarging
C6: erythrocytes circulation
C7: ventral circulation
C8: circulation toward gills
C9: circulation toward somite
M1: notochord formation
M2: somitic muscles
M3: notochord vacuolated
M4: pectoral bud
M5: cartilage apparent
M6: last pharyngeal cartilages
N1: spinal cord
N2: telencephalon
N3: myelencephalon
N4: diencephalon
N5: mesencephalon
N6: metencephalon
N7: optic chiasm
N8: optic nerve
D1: intestinal anlage
D2: liver anlage
D3: pancreas anlage
D4: esophagus anlage
D5: brush border
D6: pharynx
D7: gall bladder + bile
D8: swim bladder
D9: peristaltic undulations
D10: jaw movements
V1: optic vesicle
V2: lens
V3: marginal zone
V4: outer nuclear layer
V5: ganglion cell
V6: inner plexiform
V7: inner nuclear layer
V8: visual cell layer
V9: outer plexiform
V10: choroïdal melanocytes

## References

[1] Iwamatsu T (2004). Stages of normal development in the medaka *Oryzias latipes*. Mech Dev.

[2] Hinaux H, Pottin K, Chalhoub H, Pere S, Elipot Y, Legendre L, Retaux S (2011). A developmental staging table for *Astyanax mexicanus* surface fish and Pachon cavefish. Zebrafish.

[3] Cucchi P, Sucré E, Santos R, Leclère J, Charmantier G, Castille R (2012). Embryonic development of the sea bass *Dicentrarchus labrax*. Helgol Mar Res.

[4] Ott A, Loffler J, Ahnelt H, Keckeis H (2012). Early development of the postcranial skeleton of the pikeperch *Sander lucioperca* (Teleostei: Percidae) relating to developmental stages and growth. J Morphol.

[5] Tsai HY, Chang M, Liu SC, Abe G, Ota KG (2013). Embryonic development of goldfish (*Carassius auratus*): a model for the study of evolutionary change in developmental mechanisms by artificial selection. Dev Dyn.

[6] Kimmel CB, Ballard WW, Kimmel SR, Ullmann B, Schilling TF (1995). Stages of embryonic development of the zebrafish. Dev Dyn.

[7] Schaerlinger B, Zarski D (2015). Evaluation and improvements of egg and larval quality in percid fishes. Biology and culture of the percid fishes.

[8] Mc Elmann JF (1979). Balon EK. Early ontogeny of walleye, *Stizostedion vitreum*, with steps of saltatory development. Environ Biol Fishes.

[9] Balon EK (2002). Epigenetic processes, when *Natura Non Facit Saltum* becomes a myth, and alternative ontogenies a mechanism of evolution. Environ Biol Fishes.

[10] Balon EK (1979). The theory of saltation and its application to the ontogeny of fishes: steps and thresholds. Environ Biol Fishes.

[11] Kovac V, Copp GH (1999). Prelude: looking at early development in fishes. In when do fishes become juveniles?. Environ Biol Fishes.

[12] Peñaz M (2001). A general framework of fish ontogeny: a review of the ongoing debate. Folia Zool.

[13] Urho L (2002). Characters of larvae—what are they?. Folia Zool.

[14] Formicki K, Smaruj I, Szulc J, Winnicki A (2009). Microtubular network of the gelatinous egg envelope within egg ribbon of European perch, *Perca fluviatilis* L.. Acta Ichthyol Piscat.

[15] Korwin-Kossakowski M (2012). Fish hatching strategies: a review. Rev Fish Biol Fish.

[16] Bruslé J, Quignard JP. Biologie des poissons d’eau douce européens. Lavoisier: Tec & Doc; 2001 **(in French).**

[17] Kottelat M, Freyhof J (2007). Handbook of European freshwater fishes.

[18] Sulistyo I, Rinchard J, Fontaine P, Gardeur J, Capdeville B, Kestemont P (1998). Reproductive cycle and plasma levels of sex steroids in female Eurasian perch *Perca fluviatilis*. Aquat Living Resour.

[19] Fontaine P (2009). Développement de la pisciculture continentale européenne et domestication de nouvelles espèces. Agriculture.

[20] Zarski D, Horvath A, Kotrik L, Targonska K, Palinska K, Krejszeff S, Bokor Z, Urbanyi B, Kucharczyk D (2012). Effect of different activating solutions on the fertilization ability of Eurasian perch, *Perca fluviatilis* L., eggs. J Appl Ichtyol.

[21] Gillet C, Dubois JP, Bonnet S (1995). Influence of temperature and size of females on the timing of spawning of perch, *Perca fluviatilis*, in Lake Geneva from 1984 to 1993. Environ Biol Fishes.

[22] Sandström O, Abrahamsson I, Andersson J, Vetemaa M (1997). Temperature effects on spawning and egg development in Eurasian perch. J Fish Biol.

[23] Migaud H, Fontaine P, Sulistyo I, Kestemont P, Gardeur JN (2002). Induction of out-of-season spawning in Eurasian perch *Perca fluviatilis*. Effects of rates of cooling and cooling durations on female gametogenesis and spawning. Aquaculture.

[24] Wang N, Gardeur JN, Henrotte E, Marie M, Kestemont P, Fontaine P (2006). Determinism of the induction of the reproductive cycle in female Eurasian perch, *Perca fluviatilis*: effects of environmental cues and permissive factors. Aquaculture.

[25] Wang N, Teletchea F, Kestemont P, Milla S, Fontaine P (2010). Photothermal control of the reproductive cycle in temperate fishes. Rev Aquac.

[26] Targonska K, Szczerbowski A, Zarski D, Łuczynski MJ, Szkudlarek M, Gomułka P, Kucharczyk D (2014). Comparison of different spawning agents in artificial out-of-season spawning of Eurasian perch, *Perca fluviatilis* L.. Aquac Res.

[27] Żarski D, Krejszeff S, Horváth A, Bokor Z, Palińska K, Szentes K, Łuczyńska J, Targońska K, Kupren K, Urbányi B, Kucharczyk D (2012). Dynamics of composition and morphology in oocytes of Eurasian perch, *Perca fluviatilis* L., during induced spawning. Aquaculture.

[28] Zarski D, Palińska K, Targońska K, Bokor Z, Kotrik L, Krejszeff S, Kupren K, Horváth A, Urbányi B, Kucharczyk D (2011). Oocyte quality indicators in Eurasian perch, *Perca fluviatilis* L., during reproduction under controlled conditions. Aquaculture.

[29] Castets MD, Schaerlinger B, Silvestre F, Gardeur JN, Dieu M, Corbier C, Kestemont P, Fontaine P (2012). Combined analysis of *Perca fluviatilis* reproductive performance and oocyte proteomic profile. Theriogenology.

[30] Konstantinov KG (1957). Comparative analysis of the morphology and biology of perch, pike perch and Volga pike-perch during several developmental stages. Tr Inst Morfol Zhivoth Akad Nauk SSSR.

[31] Lereboullet A (1854). Recherches d’embryologie comparée sur le développement du brochet, de la perche et de l’écrevisse.

[32] Chevey P. Recherches sur la perche et le bar. Etude embryogénique, systématique et biogéographique des percidés européens. Bulletin biologique de la France et de la Belgique. Paris. 1925 **(in French)**.

[33] Alix M, Schaerlinger B, Ledoré Y, Chardard D, Fontaine P (2013). Developmental staging and deformities characterization of the Eurasian perch, *Perca fluviatilis*. Commun Agric Appl Biol Sci.

[34] Fontaine P, Wang N, Hemerlink B (2015). Broodstock management and control of the reproductive cycle. Biology and culture of the percid fishes.

[35] Saat T, Veersatu A (1996). The rate of early development in perch *Perca fluviatilis* L. and ruffe *Gymnocephalus cernuus* (L.) at different temperatures. Ann Zool Fenn.

[36] Gabe M (1968). Techniques histologiques.

[37] Trabelsi A, Gardeur JN, Teletchea F, Brun-Bellut J, Fontaine P (2013). Hatching time effect on the intra-spawning larval morphology and growth in Northern pike (*Esox lucius* L.). Aquac Res.

[38] Isogai S, Horiguchi M, Weinstein BM (2001). The vascular anatomy of the developing zebrafish: an atlas of embryonic and early larval development. Dev Biol.

[39] Cooper JE (1978). Eggs and larvae of the logperch, *Percina caprodes* (Rafinesque). Am Midl Nat.

[40] Kovac V (1993). Early development of the Balon’s ruff, *Gymnocephalus baloni* Holcik and Hensel, 1974. Folia Zool.

[41] Kovac V (1993). Early development of ruff, Gymnocephalus cernuus. Folia Zool.

[42] Kovac V (1992). Early development of the yellow pope, *Gymnocephalus schraester*. Folia Zool.

[43] Kovac V (1994). Early ontogeny of three Gymnocephalus species (Pisces: Percidae) reflections on the evolution of the genus. Environ Biol Fishes.

[44] Kovac V (2000). Early development of *Zingel streber*. J Fish Biol.

[45] Paine MD, Balon EK (1984). Early development of the northern logperch, *Percina caprodes semifasciata*, according to the theory of saltatory ontogeny. Environ Biol Fishes.

[46] Paine MD (1984). Balon EK Early development of the rainbow darter, *Etheostoma caeruleum*, according to the theory of saltatory ontogeny. Environ Biol Fishes.

[47] Teletchea F, Gardeur JN, Kamler E, Fontaine P (2009). The relationship of oocyte diameter and incubation temperature to incubation time in temperate freshwater fish species. J Fish Biol.

[48] Mansueti AJ (1964). Early development of the yellow perch, *Perca flavescens*. Chesap Sci.

[49] Moodie GEE, Loadman N, Wiegand MD, Mathias JA (1989). Influence of egg characteristics on survival, growth and feeding in larval walleye (*Stizostedion vitreum*). Can J Fish Aquat Sci.

[50] Wiegand MD (1996). Composition, accumulation and utilization of yolk lipids in teleost fish. Rev Fish Biol Fish.

[51] Kunz YW (2004). Developmental biology of teleost fishes.

[52] Collazo A, Bolker JA, Keller R (1994). A phylogenetic perspective on teleost gastrulation. Am Nat.

[53] Kane DA, Kimmel CB (1993). The zebrafish midblastula transition. Development.

[54] O’Boyle S, Bree RT, McLoughlin S, Grealy M, Byrnes L (2007). Identification of zygotic genes expressed at the midblastula transition in zebrafish. Biochem Biophys Res Commun.

[55] Mendelson TC, Imhoff VE, Iovine MC (2006). Analysis of early embryogenesis in rainbow and banded darters (Percidae: Etheostoma) reveals asymmetric postmating barrier. Environ Biol Fishes.

[56] Essner JJ, Amack JD, Nyholm MK, Harris EB, Yost HJ (2005). Kupffer’s vesicle is a ciliated organ of asymmetry in the zebrafish embryo that initiates left-right development of the brain, heart and gut. Development.

[57] Burggren WW (2004). What is the purpose of the embryonic heart beat? Or How facts can ultimately prevail over physiological dogma. Physiol Biochem Zool.

[58] Kvenseth AM, Pittman K, Helvik JV (1996). Eye development in Atlantic halibut (*Hippoglossus hippoglossus*): differentiation and development of the retina from early yolk sac stages through metamorphosis. Can J Fish Aquat Sci.

[59] Takashima F, Hibiya T (1995). An atlas of fish histology. Normal and pathological features.

[60] Pelluet D (1944). Criteria for the recognition of developmental stages in the salmon (*Salmo salar*). J Morphol.

[61] Kestemont P, Jourdan S, Houbarta M, Melard C, Paspatis M, Fontaine P, Cuvier A, Kentouri M, Baras E (2003). Size heterogeneity, cannibalism and competition in cultured predatory fish larvae: biotic and abiotic influences. Aquaculture.

[62] Vlavonou R. Elevage experimental de la perche *Perca fluviatilis* L.: développement larvaire et croissance. Université de Metz. 1996. p. 148 **(in French)**.

[63] Cuvier-Péres A, Kestemont P (2002). Development of some digestive enzymes in Eurasian perch larvae *Perca fluviatilis*. Fish Physiol Biochem.

[64] Cahu M, Zambobino Infante J (2007). Ontogenèse des fonctions digestives et des besoins nutritionnels chez les larves de poissons marins. Cybium.

